# Combining Photodeprotection and Ligation into a Dual‐Color Gated Reaction System

**DOI:** 10.1002/chem.202003546

**Published:** 2020-10-16

**Authors:** Jessica Alves, Tim Krappitz, Florian Feist, James P. Blinco, Christopher Barner‐Kowollik

**Affiliations:** ^1^ Centre for Materials Science Queensland University of Technology 2 George Street Brisbane QLD 4000 Australia; ^2^ School of Chemistry and Physics Queensland University of Technology 2 George Street Brisbane QLD 4000 Australia; ^3^ Max Planck Institute for Polymer Research Ackermannweg 10 55128 Mainz Germany; ^4^ Institute of Nanotechnology, INT Karlsruhe Institute of Technology Hermann-von-Helmholtz-Platz 1 76344 Eggenstein-Leopoldshafen Germany

**Keywords:** cycloaddition, Diels–Alder, photocatalysis, photooxidation, protecting groups

## Abstract

We report a photochemical reaction system which requires activation by two colors of light. Specifically, a dual wavelength gated system is established by fusing the visible light mediated deprotection of a dithioacetal with the UV light activated Diels–Alder reaction of an *o*‐methylbenzaldehyde with *n*‐ethylmaleimide. Critically, both light sources are required to achieve the Diels–Alder adduct, irradiation with visible or UV light alone does not lead to the target product. The introduced dual gated photochemical system is particularly interesting for application in light driven 3D printing, where two color wavelength activated photoresists may become reality.

## Graphical abstract

**One wavelength is not enough**: We exploit wavelength selective deprotection and photoligation, establishing a dual color gated strategy for Diels–Alder adduct formation. The visible light activated photoredox deprotection of a dithioacetal generates a UV‐light activated diene, which can be trapped by a dienophile, affording the Diels–Alder adduct only in the presence of visible and UV light.

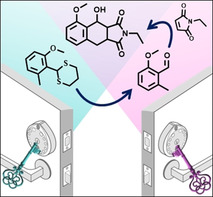

From photosynthesis, to image formation in eyes, and the camouflage of squids,[^1^, ^2^] light induced processes are complex and fascinating. In general, one of the reaction components must absorb an incident photon to induce a photochemical reaction. For example, photochemical ligations, such as the [2+2]‐cycloaddition of styrylpyrene moieties^[3]^ or the light‐induced Diels–Alder cycloaddition of *o*‐methylbenzaldehydes (*o*‐MBAs) with electron‐deficient dienophiles,^[4]^ require one reaction partner to be activated to an excited state from which the reaction can proceed.^[5]^ Apart from photochemical ligations, photosensitized reactions such as photoredox catalytic processes, are widely employed if mild reaction conditions at ambient temperatures are required.^[6]^ Through photocatalysis, a range of synthetically valuable transformations beyond simple oxidations and reductions are possible, including stereoselective reactions, cycloadditions and C−C and C−Het bond formations, as well as photocatalyzed coupling reactions, such as the Mizoroki–Heck coupling.[^7^, ^8^, ^9^, ^10^, ^11^] In order to expand the applicability of photochemical reactions, the combination of two distinct light sources, each activating different species, enables an advanced level of reaction control.

In principle, there are multiple possibilities to trigger reactions with two colors of light.^[12]^ A recent trend focuses on the development of λ‐orthogonal reactions in order to gain further reaction control. λ‐Orthogonality refers to the control over multiple reaction pathways, independently triggered by disparate wavelengths. Examples of λ‐orthogonal systems include one‐pot reaction mixtures of *o*‐methylbenzaldehydes, *N*‐ethylmaleimides and styrylpyrene moieties, where the Diels–Alder reaction can be exclusively induced in the UV region and the dimerization of the styrylpyrene only occurs under visible light irradiation.^[13]^ The major challenge in wavelength dual‐gated photochemistry is preventing the activation of the further red‐shifted photoreactive entity when shorter wavelengths are utilized, as most compounds with strong absorption in the visible region also exhibit absorbance bands extending into the UV region.

To implement this technology for applications such as advanced manufacturing, it is essential to ensure that no product is formed with a single wavelength, but is exclusively formed in the presence of both wavelengths. Direct laser writing (DLW) is a well‐established technique to print microscale 3D features onto surfaces while maintaining spatial and temporal control.^[14]^ In DLW, a liquid photoresist is cured solely in the focal point of a monochromatic light beam, provided it exhibits sufficient photon flux to enable two‐photon absorption for the reaction to proceed. Three‐dimensional structures are obtained by moving the photoresist relative to the focal point and removing any excess liquid photoresist after irradiation is complete. The high‐resolution of DLW enables novel applications of 3D printing, such as studies of cell behaviors, or in the development of electronic and photonic devices.[^15^, ^16^, ^17^] Further development on the chemistry involved in the photoresist fabrication, as proposed herein, would eliminate the need for a two‐photon absorption in order to obtain a focal point as the reactivity would be achieved only in the intersection of two laser beams.

Herein, we combine the photo‐induced deprotection of dithioacetal‐protected aldehyde moieties in the visible wavelength region,[^18^, ^19^] with *o*‐methylbenzaldehyde activation in the UV region.[^20^, ^21^, ^22^, ^23^] The mechanism of the dithioacetal deprotection, using oxidizing conditions, has been reported previously in the literature.^[19]^ While some systems have been reported for light‐mediated dual‐gated reactions, most rely on the deprotection of two protecting groups on a single molecule or on the combination of two λ‐orthogonal photoligations.[^12^, ^24^, ^25^, ^26^] To the best of our knowledge, a dual‐gated system that combines photo‐deprotection and photoligation has not yet been reported.

Pyrylium salts, such as 2,4,6‐tris(4‐methoxyphenyl)pyrylium tetrafluoroborate, are metal‐free, excited state photooxidants suitable for the deprotection of dithioacetals (Scheme 1) upon visible light irradiation.[^19^, ^27^, ^28^] When activated upon UV irradiation, the *o*‐methylbenzaldehyde forms a reactive *o*‐quinodimethane, generally referred to as “photoenol” (PE, Scheme 1). Once formed, the reactive photoenol may either form a Diels–Alder (D–A) adduct via a [4+2] cycloaddition with suitable substrates (e.g., maleimides) or rearranges back to the ground state *o*‐MBA. Alternative reaction channels can lead to the dimerization or cyclization of the photoenol moiety.[^25^, ^29^]

**Scheme 1:**
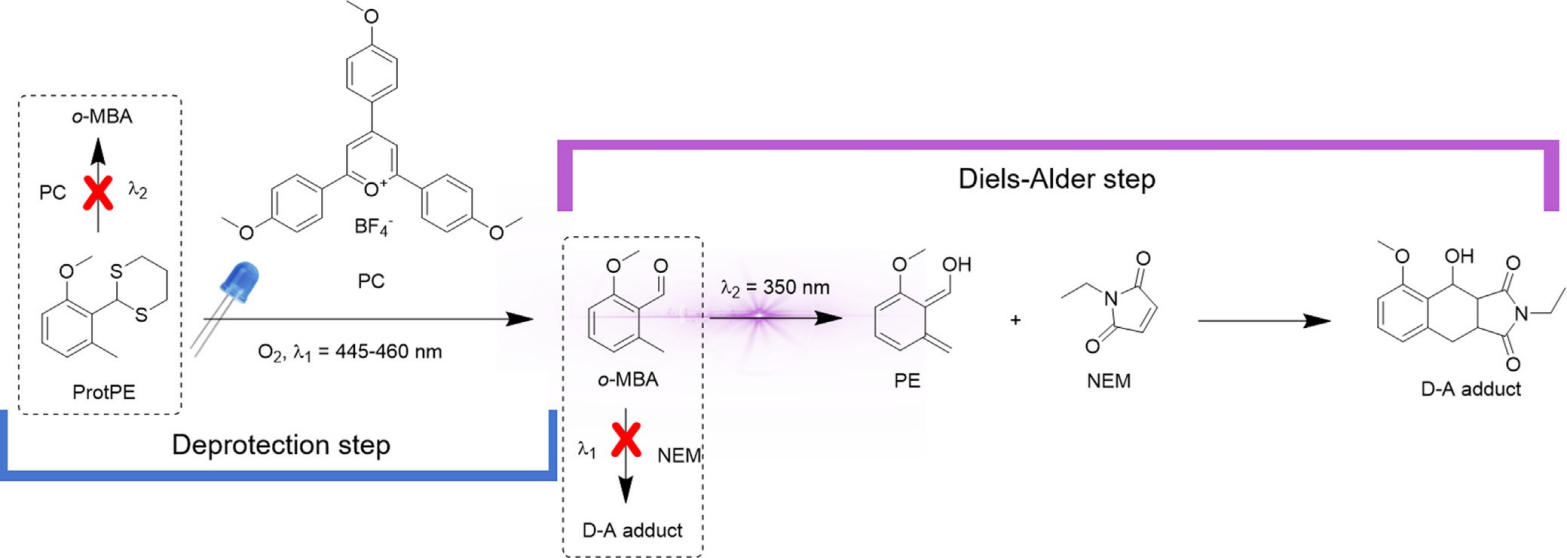
The deprotection of a dithioacetal (ProtPE) via visible light photocatalysis (PC) yields the *o*‐methylbenzaldehyde (*o*‐MBA). The *o*‐MBA rearranges to a photoenol (PE) upon UV‐light irradiation, enabling a simultaneous Diels–Alder reaction with *N*‐ethylmaleimide (NEM). This one‐pot reaction is dual color gated as the deprotection does not occur under UV irradiation and the Diels–Alder reaction does not occur under visible light irradiation.

In the following, we demonstrate the dual‐gated activation of a one‐pot reaction mixture enabling ligation exclusively in the presence of two wavelengths. The reaction mixture consists of 2‐(2‐methoxy‐6‐methylphenyl)‐1,3‐dithiane (ProtPE) as the photo caged *o*‐methylbenzaldehyde, *N*‐ethylmaleimide (NEM) as the electron poor dienophile, bicyclo[2.2.1]hept‐2‐ene as a trapping agent for reactive byproducts and 2,4,6‐tris(4‐methoxyphenyl)pyrylium tetrafluoroborate as the photoredox catalyst (PC). One of the key aspects of any dual‐gated approach is to match the kinetics of the two disparate reaction steps, that is, the deprotection step and the Diels–Alder cycloaddition. Suitable molar ratios of all reaction partners, as well as light parameters such as wavelength and intensity, need to be established in order to obtain the maximum formation of the D–A adduct. Thus, the absorption spectra and the extinction coefficients of all reaction components (Figure 1) are an important source of information. Both the *o*‐MBA as well as the Diels–Alder adduct exhibit no absorption of visible light (>400 nm), thus, an LED source (λ_1_=415–460 nm) was utilized for the activation of the photocatalyst, opening the first gate of the reaction. In order to limit competitive absorption of the UV photons by the photocatalyst, we minimized the catalyst loading to 1 mol % and utilized a monochromatic light source. Decreasing the catalyst loading further significantly slowed the deprotection step and increasing it did not have a significant improvement on the deprotection (refer to the Supporting Information section 6). Previous studies have shown that the maximum quantum yield for the Diels–Alder reaction of *o*‐MBA with *N*‐ethylmaleimide is red‐shifted compared to its maximum molar absorptivity coefficient.^[29]^ In dichloromethane, *o*‐MBA exhibits a maximum absorption at 318 nm, however we excited at 350 nm to open the second gate of the reaction by forming the photoenol, while minimizing competitive absorption.

**Figure 1:**
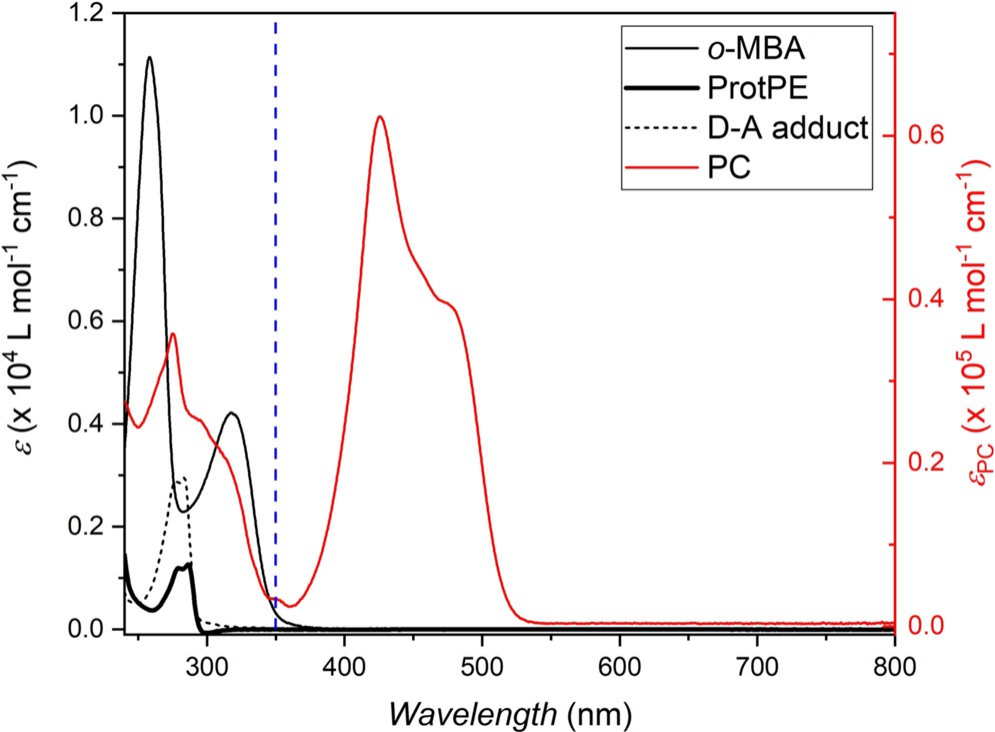
Molar extinction coefficients of *o*‐MBA, ProtPE, PC and D–A adduct in dichloromethane. At 350 nm (dashed blue vertical line) the extinction coefficients for the PC and *o*‐MBA are as follows: *ϵ*_PC_=3400±100 L mol^−1^ cm^−1^ and *ϵ*_*o*‐MBA_=315±3 L mol^−1^ cm^−1^. The ProtPE and D–A adduct exhibit no absorbance at 350 nm.

Initial attempts to conduct the wavelength dual‐gated reaction in the absence of bicyclo[2.2.1]hept‐2‐ene as a trapping reagent failed. Although the fragments from the deprotection step were not identified in the present work, it is likely that a free thiol or a disulfide is released.[^18^, ^19^, ^30^, ^31^] The disulfide is unlikely to influence the Diels–Alder reaction, however a free thiol can react in a thiol‐Michael or thiol‐ene reaction with NEM or with the reactive PE. Bicyclo[2.2.1]hept‐2‐ene is known to react more rapidly with thiols compared to maleimides (refer to the Supporting Information section 7).^[32]^ Furthermore, bicyclo[2.2.1]hept‐2‐ene is not a competitive substrate for the Diels–Alder cycloaddition of PE with NEM (refer to the Supporting Information section 8). Therefore, five equivalents of bicyclo[2.2.1]hept‐2‐ene were added to all experiments to trap any free thiols produced during the deprotection step, minimizing side reactions.^[33]^

Key experimental parameters such as the solvent, species concentrations and oxygen content have an important effect on the outcome. The Diels–Alder reaction with PEs is well examined in acetonitrile,^[29]^ while DCM is often a solvent of choice for organic photocatalysis.[^34^, ^35^] In the current study, the deprotection step was six times less efficient in acetonitrile compared to DCM (refer to the Supporting Information section 9). The decreased efficiency of the reaction in acetonitrile is hypothesized to originate from the inferior quantum yield of the PC in acetonitrile. Therefore, DCM was selected as the solvent for all experiments. The present small molecule study requires sufficiently low concentrations (i.e. 9.2 mmol L^−1^ of ProtPE) of absorbing components to achieve a homogeneous irradiation profile. In the presence of oxygen, and at low concentrations, *o*‐MBA can undergo undesired oxidation once activated by UV light. As a result, a minimal quantity of oxidative cyclized side‐product was observed in the experiments (refer to the Supporting Information section 10). Deoxygenation of the reaction mixture was not feasible as oxygen is required to recover the catalyst by closing the catalytic cycle.^[36]^

Temperature, reaction time and energy of the photons reaching the reaction mixture further influence the formation of the Diels–Alder adduct. Heat was found to favor a partial sulfoxide formation over deprotection (refer to the Supporting Information section 11). Therefore, using a low power LED for the deprotection was important in the current experimental set‐up to minimize thermal heating and afford high conversions.[^18^, ^30^, ^33^]

Evidence for the wavelength dual‐gated reaction control was obtained by performing kinetic experiments with characterization via ^1^H‐NMR spectroscopy (Figure 2 a) where all species could be quantified relative to each other (Figure 2 b). The ProtPE triplet resonance (***III***) at 7.13 ppm and the triplet resonance at 7.41 ppm (***I***), which originate from the *o*‐MBA, were used as a probe for the quantification of the respective species. The D–A adduct formation results in a distinct doublet at 5.86 ppm (***VI***), corresponding to the *α*‐proton of the hydroxy group. This resonance serves as a probe for the quantification of the D–A adduct.^[29]^ The oxidative side‐products overlap with the aromatic triplet of the D–A adduct (***II***) at 7.24 ppm, therefore, the side‐products content was determined by subtracting the known content of the D–A adduct (***VI***).

**Figure 2:**
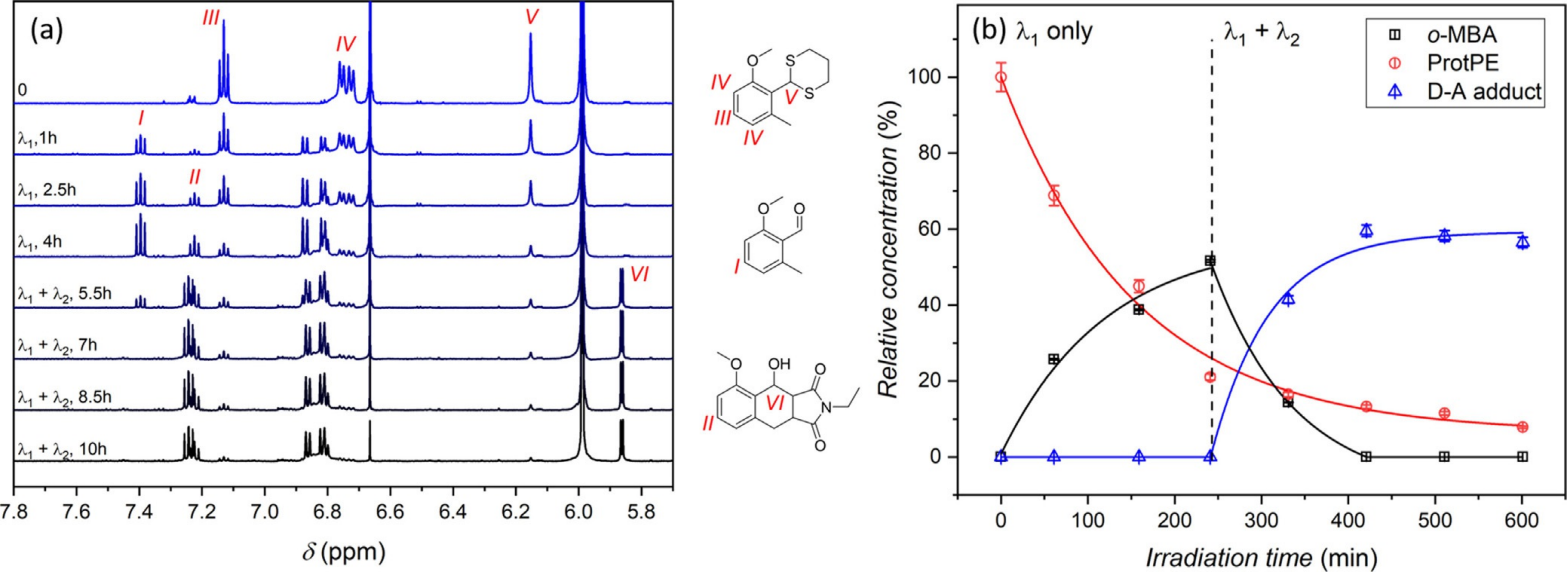
Irradiation of ProtPE (9.2 mmol L^−1^, 1 equiv), PC (1 mol %), bicyclo[2.2.1]hept‐2‐ene (5 equiv) and NEM (1.3 equiv) in 500 μL deuterated DCM with λ_1_=415 nm and subsequent additional irradiation λ_2_=350 nm. (a) ^1^H‐NMR spectra (600 MHz) of the reaction mixture upon irradiation. The resonances used to determine the conversion are the aromatic triplets for ProtPE and *o*‐MBA (*I* and *III*), the distinct doublet of the hydroxy group's α‐carbon (*VI*) for the D–A adduct and the triplet of the side‐product which overlaps with the aromatic triplet of the D–A adduct (*II*) after deducting it from the distinct doublet (*VI*) for the D–A adduct. For the full range ^1^H‐NMR spectra, refer to Supporting Information section 12. (b) Conversions determined via ^1^H‐NMR spectroscopy. The vertical dashed line indicates the transition from irradiating with λ_1_ solely to simultaneous irradiation with λ_1_ and λ_2_. Error bars were determined by the standard deviation of repeated NMR measurements and propagating the relative error to all the data points. Solid lines are a guide for the eye only.

A dual‐gated system requires formation of the desired product only when both gates are opened. Irradiating the reaction mixture only with the visible light source λ_1_, solely leads to the generation of *o*‐MBA and no Diels–Alder adduct; as the formation of photoenol requires wavelengths below 400 nm.^[29]^ To demonstrate this selectivity of our one‐pot system, the ProtPE was deprotected to approximately 80 % with λ_1_ only within 600 minutes, showing no Diels–Alder adduct formation through energy transfer processes caused by the photocatalyst. Subsequent irradiation with both light sources (λ_1_ and λ_2_) rapidly converted the *o*‐MBA to the D–A adduct, leading to approximately 60 % yield within two hours.

To further confirm the dual‐gated nature, the reaction mixture was irradiated with λ_2_ only (Figure 3 a). In this case, despite weak UV absorption no deprotection of the ProtPE occurred after 7.5 hours irradiation time, and hence no D–A adduct was formed. This is to be expected as pyrylium salts such as the photocatalyst used in this study are known to consist of two independent chromophore moieties: The 4‐arylpyrylium and the 2,6‐diarylpyrylium.^[37]^ The 2,6‐diarylpyrylium chromophore absorbs in the visible range and is responsible for enabling the catalytic process. The 4‐arylpyrylium chromophore absorbs UV light but according to literature does not induce the photoredox process. As a result, only the visible light source activates the deprotection of ProtPE. In the absence of light, no deprotection was detected after 17 hours.

**Figure 3:**
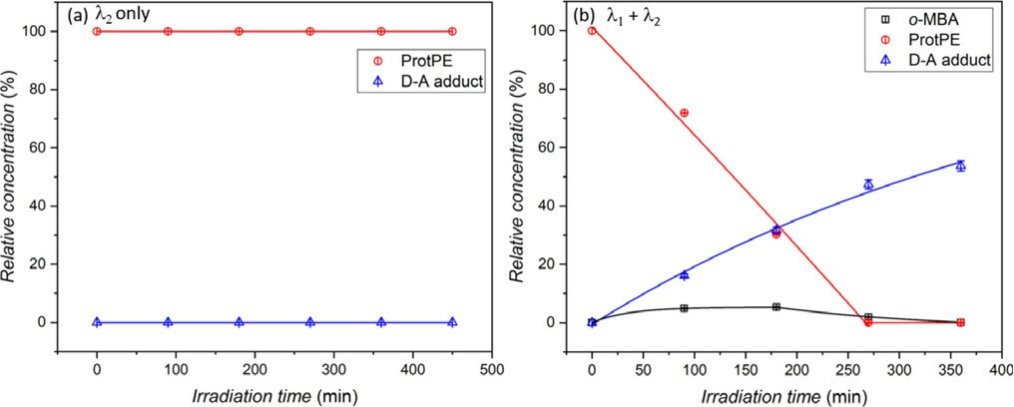
Irradiation of ProtPE (9.2 mmol L^−1^, 1 equiv), PC (1 mol %), bicyclo[2.2.1]hept‐2‐ene (5 equiv) and NEM (1.3 equiv) in 500 μL deuterated DCM with (a) only λ_2_=350 nm and (b) λ_1_=415 nm and λ_2_ simultaneously. Conversions determined via ^1^H‐NMR spectroscopy from the aromatic triplets for ProtPE and *o*‐MBA and the distinct doublet of the hydroxy group's α‐carbon for the D–A adduct. Error bars were determined by the standard deviation of repeated NMR measurements and propagating the relative error to all the data points. Solid lines are a guide for the eye only.

To further explore the robustness of the dual‐gated reaction, the reaction mixture was simultaneously irradiated with both wavelengths (Figure 3 b). The conversions obtained by using either simultaneous or subsequent irradiation were found to be similar. In both approaches, simultaneous and sequential, the D–A adduct plateaus at about 50–60 % yield. The two experiments highlight once more the requirement for both wavelengths to be present to induce the dual‐gated reaction, enabling additional control compared to single wavelength photochemistry.

In summary, we introduce a photochemical reaction system that requires the presence, subsequent or simultaneously, of two disparate wavelengths of light to form a Diels–Alder adduct. No cross reactivity was observed for the two underlying reaction steps and each of the gates were only opened with the key wavelength. Hence, we developed a wavelength dual‐gated system which is of key interest for the development of dual color photoresists.

## Conflict of interest

The authors declare no conflict of interest.

## Supporting information

As a service to our authors and readers, this journal provides supporting information supplied by the authors. Such materials are peer reviewed and may be re‐organized for online delivery, but are not copy‐edited or typeset. Technical support issues arising from supporting information (other than missing files) should be addressed to the authors.

Supplementary
