# Supplementary material for: Combining Photodeprotection and Ligation into a Dual‐Color Gated Reaction System
Source: Chemistry. 2020 Oct 16;26(71):16985–9. doi: 10.1002/chem.202003546 (PMC7894288; doi:10.1002/chem.202003546)
Supplement: Supplementary file 1 — Supplementary [file CHEM-26-16985-s001.pdf]

Chemistry–A European Journal

Supporting Information

Combining Photodeprotection and Ligation into a Dual-Color Gated Reaction System

Jessica Alves,[a, b] Tim Krappitz,[a, b] Florian Feist,[a, b, c] James P. Blinco,*[a, b] and Christopher Barner-Kowollik*[a, b, d]

Content

1 Materials and General Procedure 2

1.1 Materials 2

1.2 General Procedure for Wavelength Dual-Gated Experiments 2

2 Instrumentation 3

2.1 Flash Column Chromatography 3

2.2 UV-Vis Spectrometer 3

2.3 Nuclear Magnetic Resonance Spectroscopy (NMR) 3

2.4 Laser Experiments 3

2.5 Liquid Chromatography Coupled with Mass Spectrometry (LC-HRMS) 4

3 Synthesis 5

3.1 2-methoxy-6-methylbenzaldehyde (*o*-MBA) 5

3.2 2-(2-methoxy-6-methylphenyl)-1,3-dithiane (ProtPE) 6

3.3 2-ethyl-4-hydroxy-5-methoxy-3a,4,9,9a-tetrahydro-1H-benzo[f]isoindole-1,3(2H)-dione (D-A adduct) 7

4 NMR Spectra of the Substrates in CD_2Cl_2 8

4.1 2-methoxy-6-methylbenzaldehyde (*o*-MBA) 8

4.2 2-(2-methoxy-6-methylphenyl)-1,3-dithiane (ProtPE) 9

4.3 2,4,6-tris(4- methoxyphenyl)pyrylium tetrafluoroborate (PC) 10

4.4 2-ethyl-4-hydroxy-5-methoxy-3a,4,9,9a-tetrahydro-1H-benzo[f]isoindole-1,3(2H)-dione (D-A Adduct) 10

5 Emission Spectrum of LEDs 11

6 Catalyst loading 12

7 Thiol-ene Reaction with Bicyclo[2.2.1]hept-2-ene and NEM 13

8 Orthogonality of Bicyclo[2.2.1]hept-2-ene and *o*-MBA under Irradiation 15

9 ProtPE Deprotection in Acetonitrile 15

10 Cyclisation of *o*-MBA under UV Light Irradiation 16

11 Heat Effect on the Deprotection of ProtPE 20

12 Full ^{1}H-NMR Spectra of Sequential Irradiation 22

13 Maximum Deprotection 23

14 Maximum Diels-Alder Adduct Formation 24

15 References 26

1 Materials and General Procedure

1.1 Materials

2,3-Dimethyl anisole (2944-49-2, Combi-blocks), copper (II) sulphate pentahydrate ($CuSO_4 \cdot 5H_2O$) (7758-99-8, VWR International), potassium peroxodisulfate ($K_2S_2O_8$) (7727-21-1, Merck), magnesium sulfate anhydrous ($MgSO_4$) (7487-88-9, Merck), 1,3-propanedithiol (109-80-8, Sigma-Aldrich), iodine (7553-56-2, Sigma-Aldrich), sodium thiosulfate ($Na_2S_2O_3$) (7772-98-7, Fisher Scientific), sodium hydroxide (NaOH) (1310-73-2, Chem-Supply), *N*-ethylmaleimide (NEM) (128-53-0, Sigma-Aldrich), Aluminium oxide (Alox) (activated, basic, Brockmann I) (1344-28-1, Thermo Fisher), bicyclo[2.2.1]hept-2-ene (498-66-8, Sigma Aldrich), 1,3,5-trimethoxybezene (TMB) (621-23-8, Sigma-Aldrich), dichloromethane (75-09-2, Thermo Fisher), ethyl acetate (EtOAC) (141-78-6, Thermo Fisher), cyclohexane (108-94-1, Thermo Fisher), chloroform-*d* (99.8 %D, Cambridge Isotope Laboratories) and methylene chloride-d_2 (99.9 %D, Sigma-Aldrich) were used as received. The photocatalyst (PC) 2,4,6-tris(4-methoxyphenyl)pyrylium tetrafluoroborate was synthesized according to a previously reported procedure.[1]

1.2 General Procedure for Wavelength Dual-Gated Experiments

Unless otherwise stated, the irradiation experiments were performed in an NMR tube with methylene chloride-d_2 as a solvent. A typical reaction mixture consisted of 2-(2-methoxy-6-methylphenyl)-1,3-dithiane (ProtPE, 1.00 eq, 9.2 μmol·mL^{-1}), *N*-ethylmaleimide (NEM, 1.30 eq) as dienophile, bicyclo[2.2.1]hept-2-ene (5.00 eq) as trapping agent and the photocatalyst (PC, 1mol%). Stock solutions were prepared for the photocatalyst as well as bicyclo[2.2.1]hept-2-ene in order to guarantee accurate measurements. The reaction volume for all experiments was 500 μL. The visible light irradiation was performed with a 3W LED (λ_{max} = 445 nm) in 25 mm distance from the side of the NMR tube. A nanosecond pulsed laser beam tuned to 350 nm (approximately 70 μJ per pulse, 20 Hz, directed upwards toward the bottom of the NMR tube) was used for the ultra-violet irradiation. The irradiation time was controlled by an external shutter regulated with an Arduino-board. ^{1}H-NMR spectra were recorded at set times.

2 Instrumentation

2.1 Flash Column Chromatography

Flash chromatography was performed on an *Interchim* XS420+ flash chromatography system consisting of a SP-in-line filter 20-µm, an UV-VIS detector (200-800 nm). The separations were performed using an *Interchim* dry load column and an *Interchim* Puriflash Silica HP 30 µm column. The crude materials were deposited on celite 545 prior to chromatography.

2.2 UV-Vis Spectrometer

UV-Vis spectra were recorded on a *Shimadzu* UV-2700 spectrophotometer equipped with a CPS-100 electronic temperature control cell positioner. Samples were prepared in dichloromethane and measured in *Hellma Analytics* quartz high precision cell cuvettes at 20 °C.

2.3 Nuclear Magnetic Resonance Spectroscopy (NMR)

^{1}H-NMR spectra were recorded on a Bruker System 600 Ascend LH, equipped with a BBO-Probe (5 mm) with z-gradient (^{1}H: 600.13 MHz, ^{13}C: 150.90 MHz). All measurements were carried out in deuterated solvents. The chemical shift (δ) is recorded in parts per million (ppm) and relative to the residual solvent protons. Coupling constants (J) were calculated in Hertz (Hz). *MestReNova* 12.0 software was used to analyze the spectra. The signals were quoted as follows: s = singlet, bs =broad singlet, d = doublet, dd = doublet of doublets, t = triplet and m = multiplet.

2.4 Laser Experiments

The laser experiments were carried out according to the following procedure (Scheme S1): the incident light used for laser experiments was a Coherent Opolette 355 tunable OPO with a pulse full width at half maximum of 7 ns and a repetition rate of 20 Hz. The emitted pulse, which has a flat-top spatial profile, was expanded to 6 mm diameter using focusing lenses and directed upwards using a prism. The energy was adjusted using the Q-Switch delay. The energy transmitted through the sample holder was measured using a Coherent Energy Max PC power meter. The visible irradiation was performed from the side while the laser irradiation came from below.

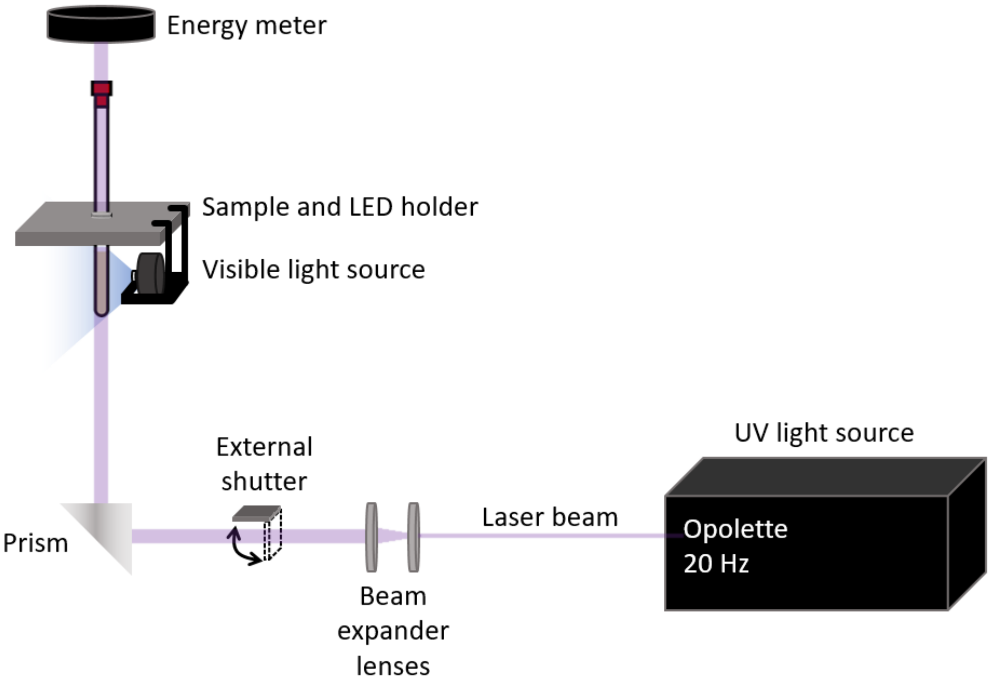

Scheme S1. Experimental setup for the experiments with two light-sources.

2.5 Liquid Chromatography Coupled with Mass Spectrometry (LC-HRMS)

LC-HRMS measurements were performed on an UltiMate 3000 UHPLC System (Dionex, Sunnyvale, CA, USA) consisting of a pump (LPG 3400SZ), autosampler (WPS 3000TSL) and a temperature-controlled column compartment (TCC 3000, 40 °C). A C18 HPLC column (Phenomenex Luna 5µm, 100 Å, 250 × 2.0 mm) was utilized for separation. Water (containing 5 mmol·L^{-1} ammonium acetate) and acetonitrile were used as eluents. A gradient of ACN:H_2O 5:95 to 100:0 (v/v) in 7 min at a flow rate of 0.40 mL·min^{-1} was applied. The flow was split in a 9:1 ratio, where 90 % of the eluent was directed through a DAD UV-detector (VWD 3400, Dionex) and 10 % was infused into the electrospray source. Spectra were recorded on an LTQ Orbitrap Elite mass spectrometer (Thermo Fisher Scientific, San Jose, CA, USA) equipped with a HESI II probe. The instrument was calibrated in the m/z range 74-1822 using premixed calibration solutions (Thermo Scientific). A constant spray voltage of 3.5 kV, a dimensionless sheath gas and a dimensionless auxiliary gas flow rate of 5 and 2 were applied, respectively. The capillary temperature and was set to 300 °C, the S-lens RF level was set to 68, and the aux gas heater temperature was set to 100 °C.

3 Synthesis

3.1 2-methoxy-6-methylbenzaldehyde (*o*-MBA)

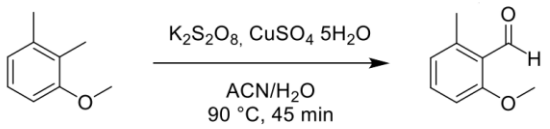

2-methoxy-6-methylbenzaldehyde was synthesized using a modified literature procedure.[2,3] 2,3-Dimethyl anisole (6.9 g, 7 mL, 50.6 mmol, 1.00 eq), $CuSO_4 \cdot 5H_2O$ (13.33 g, 53.4 mmol, 1.06 eq) and $K_2S_2O_8$ (41.97 g, 155.2 mmol, 3.07 eq) were added to a 1:1 acetonitrile/water mixture (500 mL) and vigorously stirred at 90 °C for 1 hour, until the TLC showed complete consumption of the starting material. Afterwards, the mixture was cooled to room temperature and filtered to remove the undissolved copper salts. Water (50 mL) was added to the reaction mixture and the aqueous phase was extracted with DCM (3 x 150 mL). The combined organic layers were dried over $MgSO_4$, filtered, and the solvent was removed under reduced pressure. The obtained brown oil was purified *via* sublimation for 12 hours under inert atmosphere at 90 °C yielding 2.0 g (26 %) of 2-methoxy-6-methylbenzaldehyde (*o*-MBA) as a colorless crystalline solid. ^1H and ^{13}C- NMR spectra are identical to literature reports.[4]

^{1}H NMR (600 MHz, Chloroform-*d*): δ = 10.66 (s, 1H, CHO), 7.38 (t, J = 7.8 Hz, 1H, Ar H), 6.84 (d, J = 8.3, 1H), 6.81 (d, J = 7.6 Hz, 1H), 3.89 (s, 3H, OCH_3), 2.57 (s, 3H, CH_3) ppm.

^{13}C NMR (151 MHz, Chloroform-*d*): δ = 192.39, 163.25, 142.07, 134.54, 124.16, 123.38, 109.13, 55.83, 21.53.

LC-HRMS, refer to Figure S1.

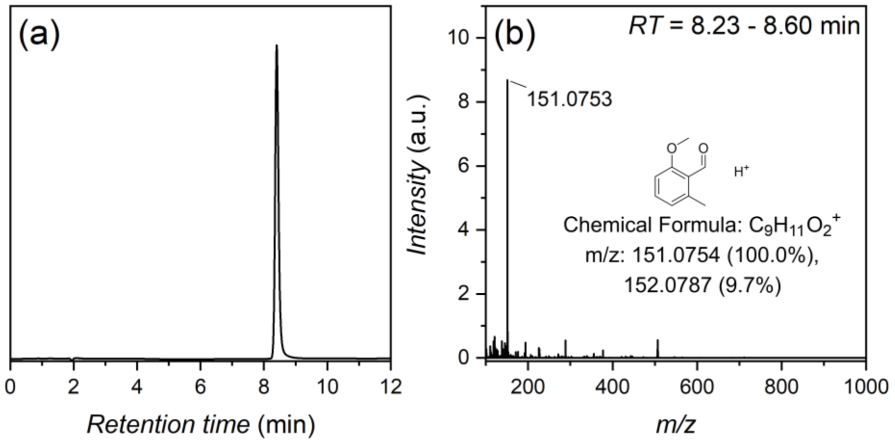

Figure S1. LC-HRMS analysis of the *o*-MBA. (a) Chromatogram of the 254 nm channel and (b) Accumulated MS spectra at *RT* = 8.23 – 8.60 min with the insert of the chemical structure and its m/z prediction (0.7 ppm deviation).

3.2 2-(2-methoxy-6-methylphenyl)-1,3-dithiane (ProtPE)

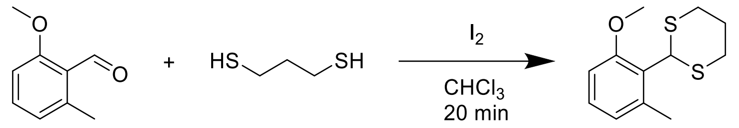

The synthetic procedure was adapted from the literature.[5] A solution of 0.69 mmol (100.46 mg, 1.00 eq) of 2-methoxy-6-methylbenzaldehyde (*o*-MBA) in 5 mL $CHCl_3$ was mixed with 0.74 mmol (73.7 μL, 1.10 eq) 1,3-propanedithiol and 0.07 μmol (17 mg, 0.10 eq) iodine. The mixture was stirred at room temperature for 20 minutes. The reaction was quenched with aqueous solutions of $Na_2S_2O_3$ (5 mL, 0.1 M) and NaOH (5 mL, 1 M). The organic phase was separated, and the aqueous phase extracted 3x5 mL $CHCl_3$. The combined organic phases were dried over $MgSO_4$ and filtered. The solvent was removed under reduced pressure yielding 141 mg (87% yield) of 2-(2-methoxy-6-methylphenyl)-1,3-dithiane (ProtPE) as a yellowish solid.

^{1}H NMR (600 MHz, Chloroform-*d*): δ = 7.12 (t, *J* = 7.9 Hz, 1H), 6.73 (dd, *J* = 36.2, 7.8 Hz, 2H), 6.19 (s, 1H), 3.85 (s, 3H), 3.10 (t, *J* = 12.3 Hz, 2H), 2.88 (broad, 2H), 2.75 (s, 3H), 2.21 – 2.11 (m, 1H), 1.97 – 1.87 (m, 1H).

^{13}C NMR (151 MHz, Chloroform-*d*): δ = 156.07, 140.72, 128.87, 125.34, 124.63, 108.15, 56.16, 44.14, 32.81, 25.79, 20.93.

LC-HRMS, refer to Figure S2.

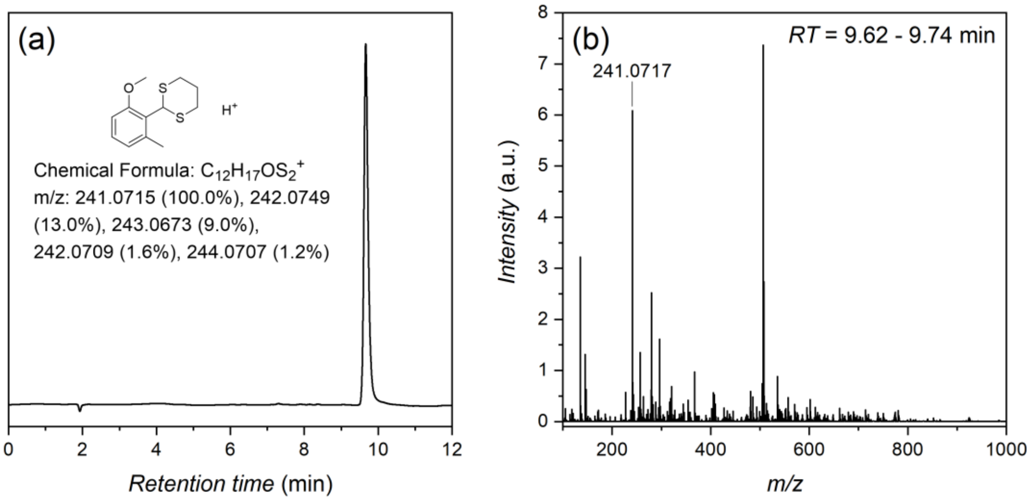

Figure S2. LC-HRMS analysis of the ProtPE. (a) Chromatogram of the 254 nm UV-Vis channel with the insert of the chemical structure and its m/z prediction and (b) Accumulated MS spectra at *RT* = 9.62 – 9.74 min. The mass deviation is of 0.8 ppm.

3.3 2-ethyl-4-hydroxy-5-methoxy-3a,4,9,9a-tetrahydro-1H-benzo[f]isoindole-1,3(2H)-dione (D-A adduct)

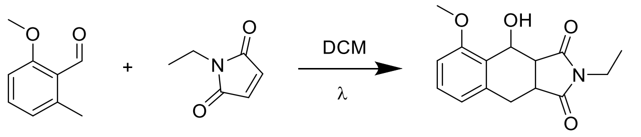

A solution of 67 µmol (20 mg, 1 eq) of 2-methoxy-6-methylbenzaldehyde (*o*-MBA) in 1 mL DCM was mixed with 87 µmol (11 mg, 1.3 eq) *N*-ethylmaleimide. The solution was deoxygenated by passing through a stream of nitrogen. The mixture was stirred at ambient temperature under irradiation with a LED (365 nm, 3W, 1 cm below the reaction mixture) for 4 hours. The Diels-Alder adduct was purified *via* flash chromatography using EtOAc and cyclohexane as mobile phases (CH:EE 100:0 – 80:20 v/v) giving 5 mg (27 % yield) of the D-A adduct as a white solid. The NMR spectra of the D-A adduct are similar to ones described in the literature but recorded in different deuterated solvents.[2]

^{1}H NMR (600 MHz, Methylene chloride-d_2) δ 7.24 (t, *J* = 7.4 Hz, 1H), 6.86 (d, *J* = 7.5 Hz, 1H), 6.81 (d, *J* = 8.3 Hz, 1H), 5.85 (d, *J* = 4.2 Hz, 1H), 3.82 (s, 3H), 3.62 – 3.52 (m, 2H), 3.17 – 3.06 (m, 3H), 2.96 (m, 1H), 1.16 (t, *J* = 7.2 Hz, 3H).

^{13}C NMR (151 MHz, Methylene chloride-d_2) δ 180.22, 177.51, 156.54, 138.99, 130.12, 126.45, 121.33, 109.89, 61.76, 56.14, 47.15, 38.78, 34.11, 28.19, 13.30.

LC-HRMS, refer to Figure S3.

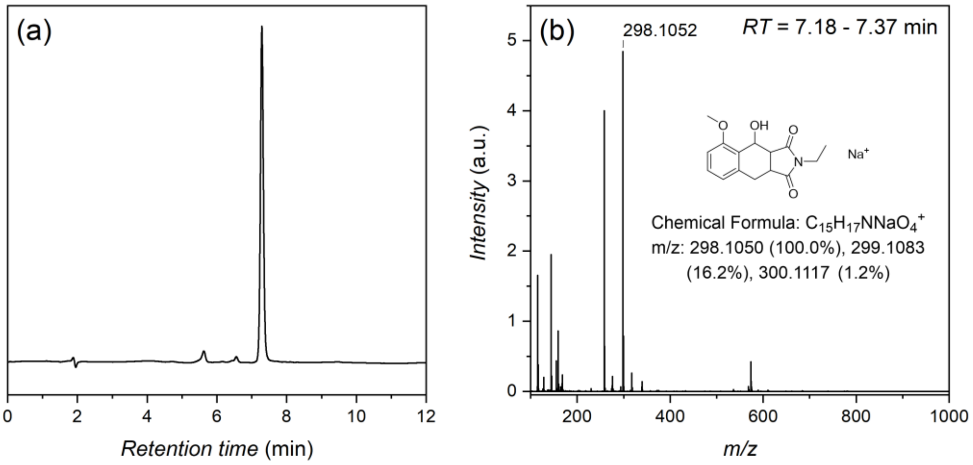

Figure S3. LC-HRMS analysis of the Diels-Alder adduct. (a) Chromatogram of the 254 nm UV-Vis channel and (b) Accumulated MS spectra at *RT* = 7.18 – 7.37 min with the insert of the chemical structure and its m/z prediction (0.7 ppm deviation).

4 NMR Spectra of the Substrates in CD_2Cl_2

4.1 2-methoxy-6-methylbenzaldehyde (*o*-MBA)

^{1}H NMR (600 MHz, Methylene Chloride-d_2): δ = 10.62 (1, s, 1H), 7.40 (2, t, *J* = 8.4, 7.6 Hz, 1H), 6.87 (3, d, *J* = 8.4 Hz, 1H), 6.81 (4, d, *J* = 7.8 Hz, 1H), 3.89 (5, s, 4H), 2.54 (6, s, 3H).

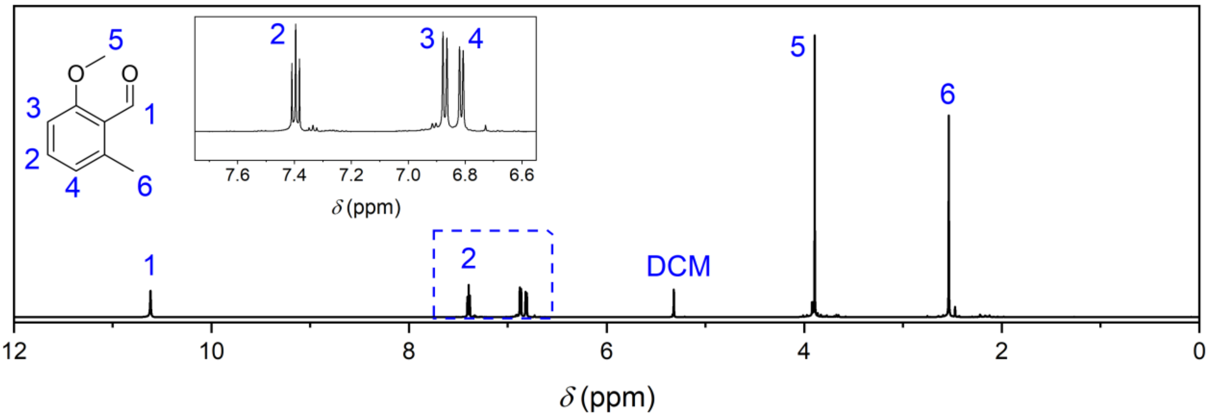

Figure S4. ^{1}H NMR spectrum (600 MHz) of *o*-MBA in CD_2Cl_2 and assigned resonances.

^{13}C NMR (151 MHz, Methylene Chloride-d_2): δ = 192.52, 163.77, 142.26, 134.91, 124.50, 123.95, 109.75, 56.35, 21.68.

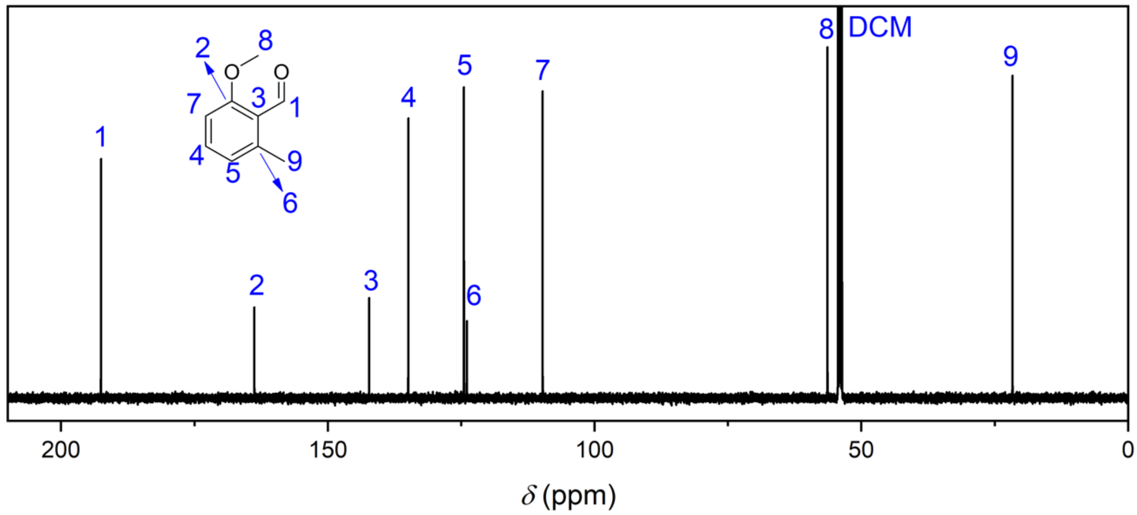

Figure S5. ^{13}C NMR spectrum (151 MHz) of *o*-MBA in CD_2Cl_2 and assigned resonances.

4.2 2-(2-methoxy-6-methylphenyl)-1,3-dithiane (ProtPE)

^{1}H NMR (600 MHz, Methylene Chloride-d_2): δ = 7.14 (1, t, J = 7.9 Hz, 1H), 6.75 (2, 3, dd, J = 18.0, 7.9 Hz, 2H), 6.16 (4, s, 1H), 3.85 (5, s, 3H), 3.10 (7, m, 2H), 2.87 (8, m, 2H), 2.72 (6, s, 3H), 2.17 (9, m, 1H), 1.87 (10, m, 1H).

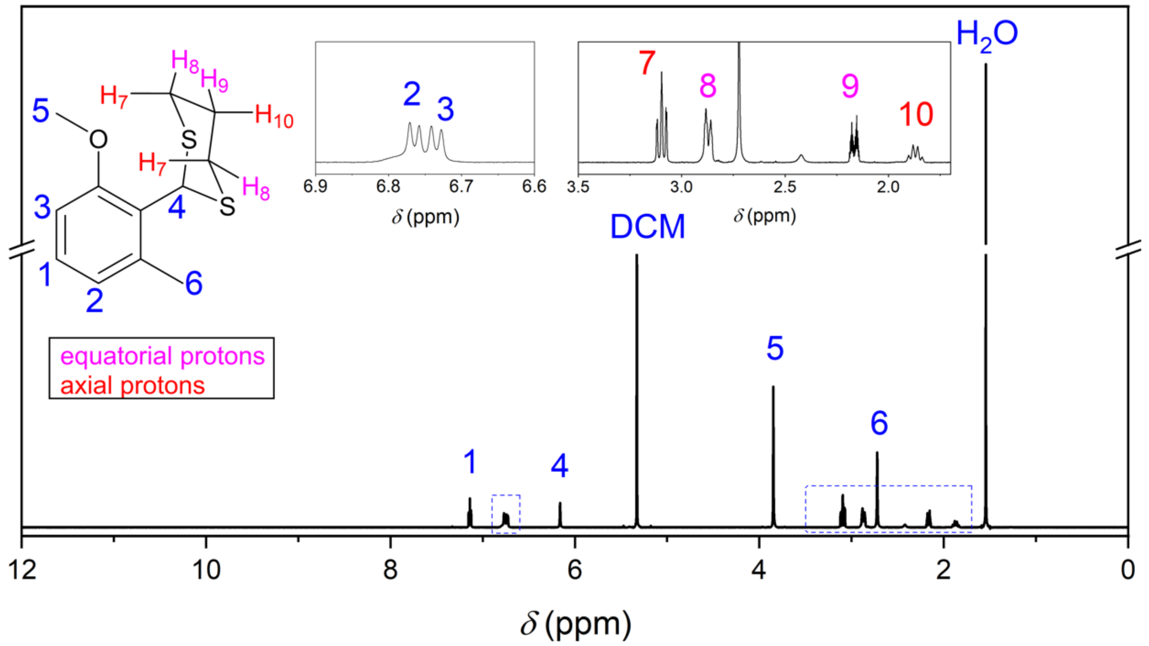

Figure S6. ^{1}H NMR spectrum (600 MHz) of ProtPE in CD_2Cl_2 and assigned resonances.

4.3 2,4,6-tris(4- methoxyphenyl)pyrylium tetrafluoroborate (PC)

^{1}H NMR (600 MHz, Methylene chloride-d_2): δ = 8.33 – 8.17 (1, 2, m, 8H), 7.28 – 7.16 (3, m, 6H), 3.99 (4, s, 9H).

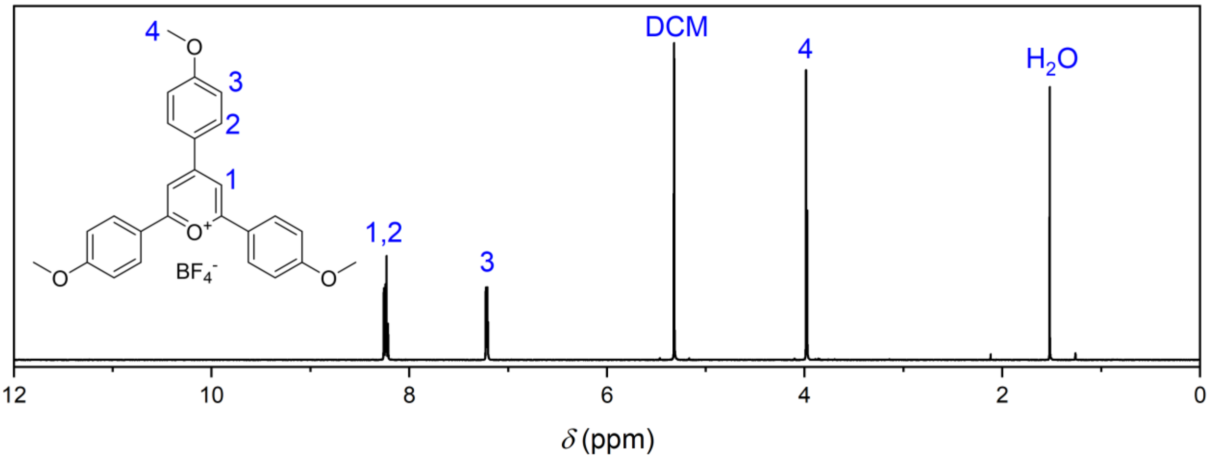

Figure S7. ^{1}H NMR spectrum (600 MHz) of PC in CD_2Cl_2 and assigned resonances.

4.4 2-ethyl-4-hydroxy-5-methoxy-3a,4,9,9a-tetrahydro-1H-benzo[f]isoindole-1,3(2H)-dione (D-A Adduct)

^{1}H NMR (600 MHz, Methylene chloride-d_2) δ 7.24 (1, t, *J* = 7.4 Hz, 1H), 6.86 (2, d, *J* = 7.5 Hz, 1H), 6.81 (3, d, *J* = 8.3 Hz, 1H), 5.85 (4, d, *J* = 4.2 Hz, 1H), 3.82 (5, s, 3H), 3.62 – 3.52 (6, m, 2H), 3.17 – 3.06 (7, 8, m, 3H), 2.96 (9, m, 1H), 1.16 (10, t, *J* = 7.2 Hz, 3H).

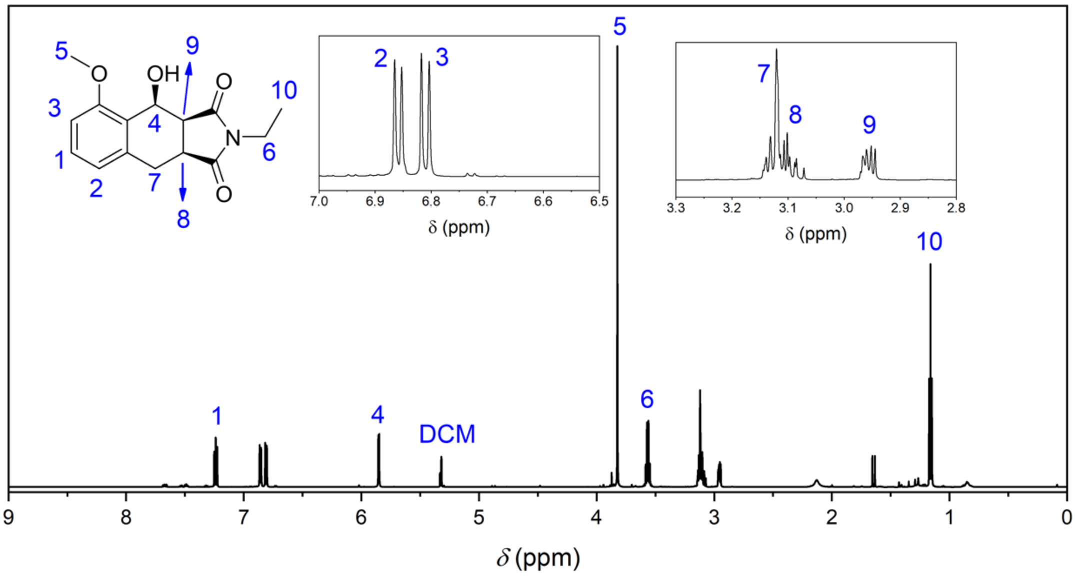

Figure S8. ^{1}H NMR spectrum (600 MHz) of Diels-Alder adduct in CD_2Cl_2 and assigned resonances.

^{13}C NMR (151 MHz, Methylene chloride-*d*$_2$) δ 180.22, 177.51, 156.54, 138.99, 130.12, 126.45, 121.33, 109.89, 61.76, 56.14, 47.15, 38.78, 34.11, 28.19, 13.30.

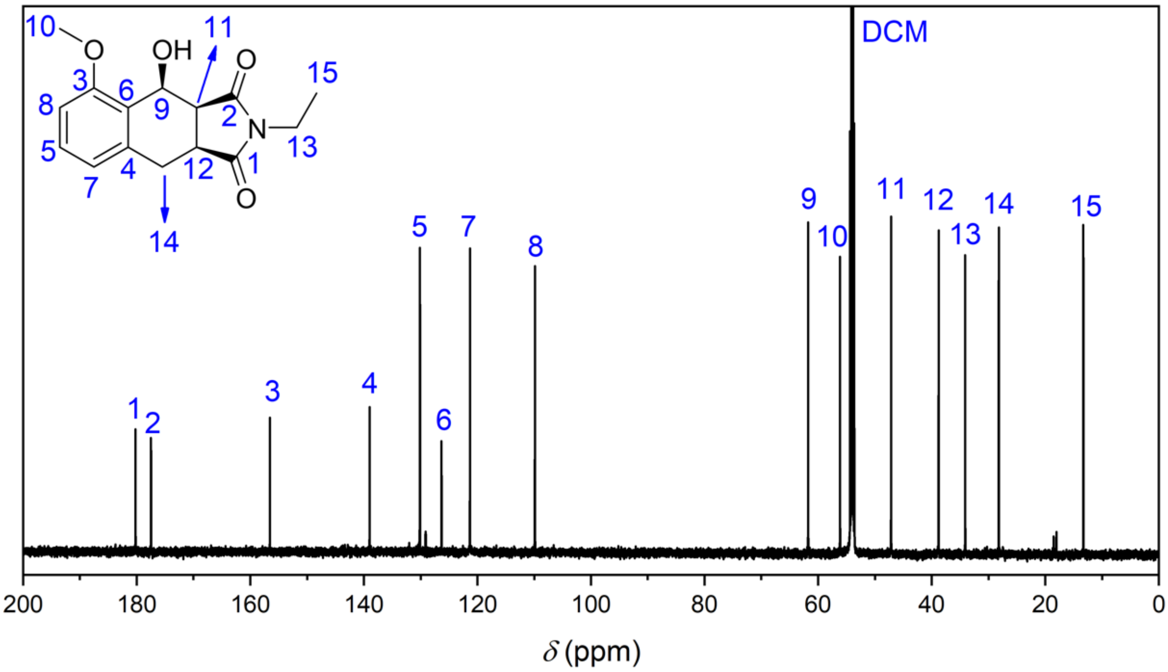

Figure S9. ^{13}C NMR spectrum (151 MHz) of Diels-Alder adduct in CD_2Cl_2 and assigned resonances.

5 Emission Spectrum of LEDs

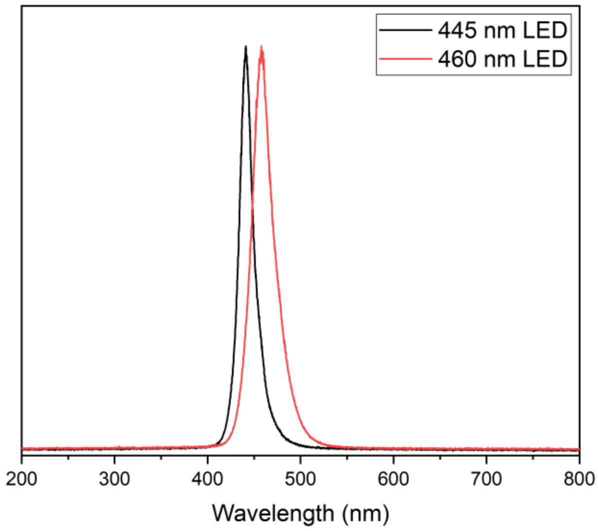

Figure S10. Normalized intensity of the emission spectrum of the blue LEDs used for the deprotection (λ_{max} = 445 nm, 10 W and λ_{max} = 460 nm, 3 W). The full width at half maximum (FWHM) of the 460 nm LED is 26 nm while for the 445 nm is 17 nm. The 460 nm, 3W LED emits from 445 to 470 nm and the 445 nm LED emits from 430 to 450 nm.

6 Catalyst loading

0.44—0.55 mg ProtPE (9—9.2 mmol·L^{-1}), with different catalyst loadings (2mol%, 1mol%, 0.1mol%) PC and 3 eq NEM were dissolved in 250 μL deuterated dichloromethane each. The reaction mixture was transferred into a crimped 0.7 mL photovial by LLG Labware, Lab Logistic Group GmbH (Art. Nr. 4-008202) equipped with a magnetic stir bar. The experiment was performed under vigorous stirring following the general standard procedure described in section 1.2. From Figure S11, a full conversion of ProtPE to o-MBA is observe within 60min at 1mol% catalyst loading. With ten times less PC (0.1mol% catalyst loading), it is estimated that only 66% ProtPE was converted in the same time period.

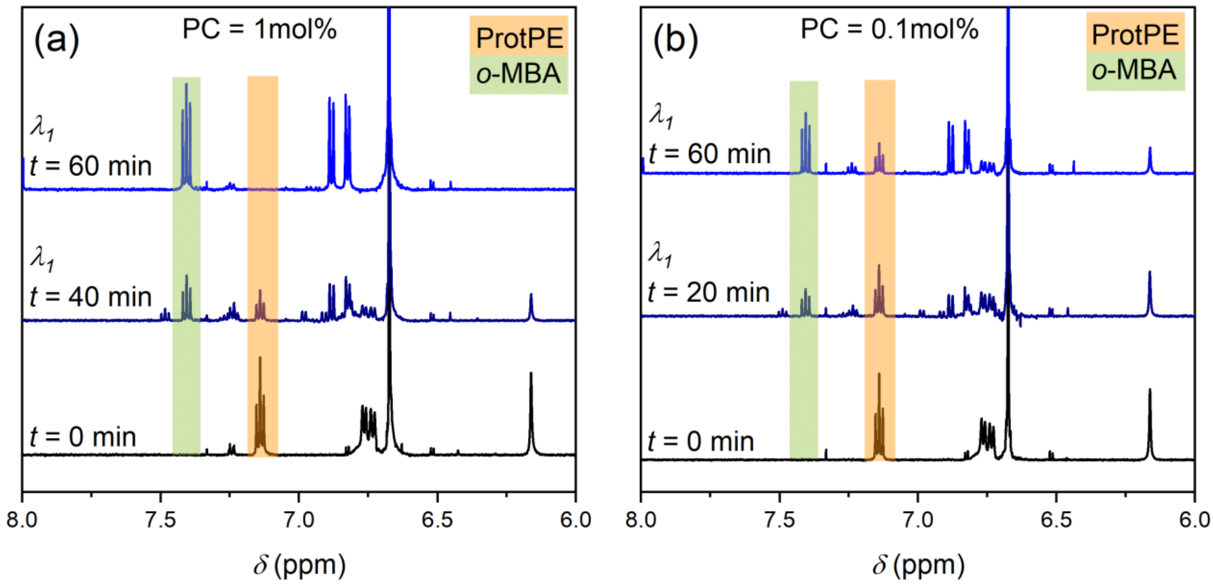

Figure S11. Study on the kinetics of deprotection dependent on the quantity of photocatalyst (PC) in solution. (a) With 1mol% PC, full deprotection is reached after 60 min irradiation with λ_1 = 460 nm. The laser vial was irradiated at a distance of 45 mm from the side of the vial. (b) with 0.1mol% catalyst loadingPC, only 66% conversion was observed after 60 min irradiation with same light source placed in a distance of 25 mm from the vial.

In comparison, increasing the photocatalyst loading from 1mol% to 2mol% causes no significant difference in the kinetics (Figure S12).

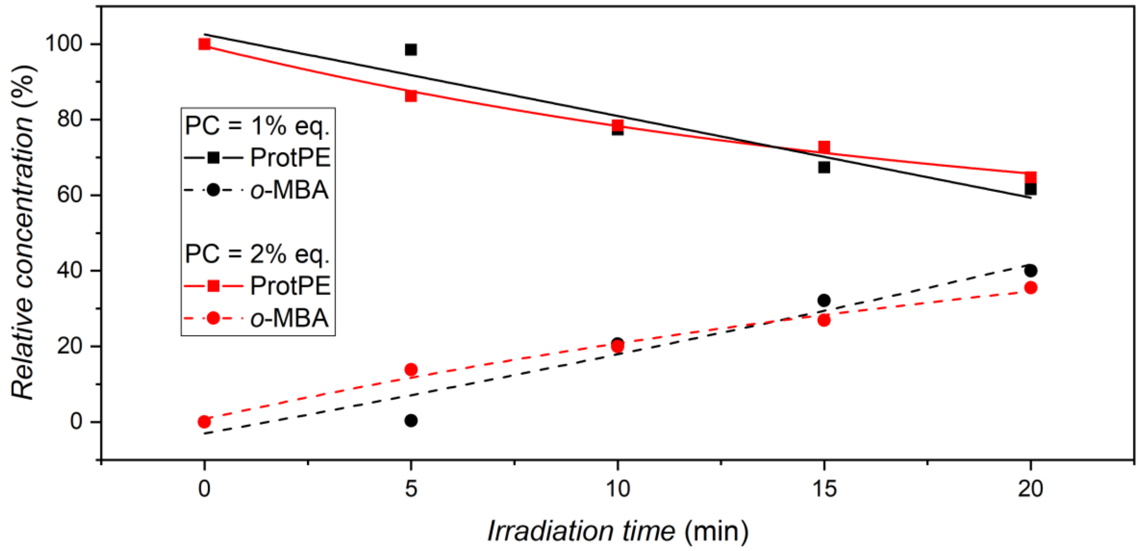

Figure S12. Analysis on the conversion of ProtPE to *o*-MBA under irradiation with λ_1 = 460 nm with a varying PC loading. In this experiment, 1.00 eq 1,3,5-trimethoxybenzene was added to the stock solutions in order to provide a NMR standard to ease determination of the concentrations.

7 Thiol-ene Reaction with Bicyclo[2.2.1]hept-2-ene and NEM

140 µmol of bicyclo[2.2.1]hept-2-ene or NEM was added to 14 µL (1 eq) 1,3-propanedithiol in 500 µL deuterated dichloromethane. The samples were kept in the dark and ^{1}H NMR spectra were measured over time. In the bicyclo[2.2.1]hept-2-ene experiment, the resonance at 6 ppm, which corresponds to the alkene protons, disappeared quickly, indicating that full conversion was achieved in less than 3 hours (Figure S13).

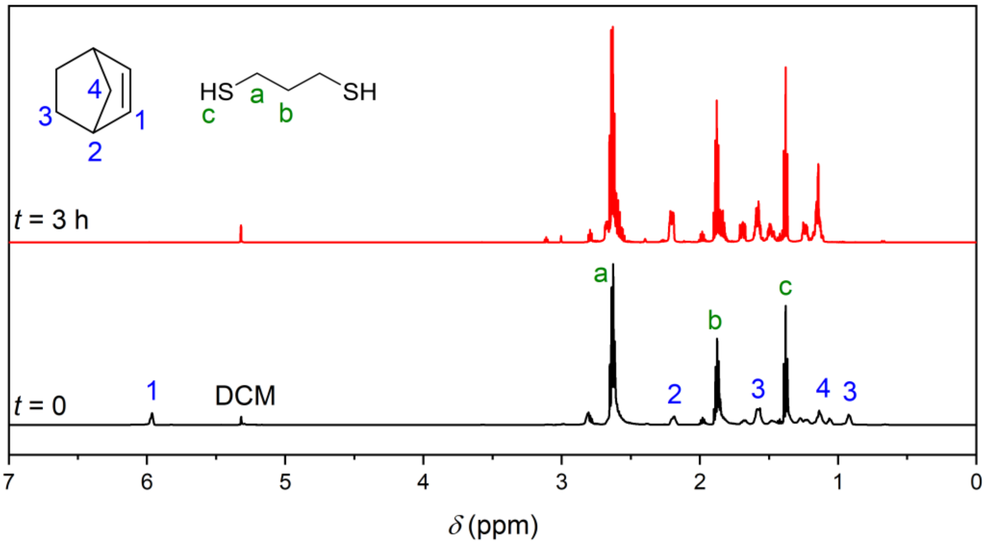

Figure S13. ^{1}H NMR of a mixture of bicyclo[2.2.1]hept-2-ene and 1,3-propanedithiol in CD_2Cl_2 at t=0 and after 3 hours at ambient temperature showing full conversion of norbonene, evident from the vanishing of the alkene protons resonances of bicyclo[2.2.1]hept-2-ene at δ = 5.9 ppm.

When NEM is used instead of bicyclo[2.2.1]hept-2-ene, no reaction was observed after 24 hours at ambient temperature (Figure S14).

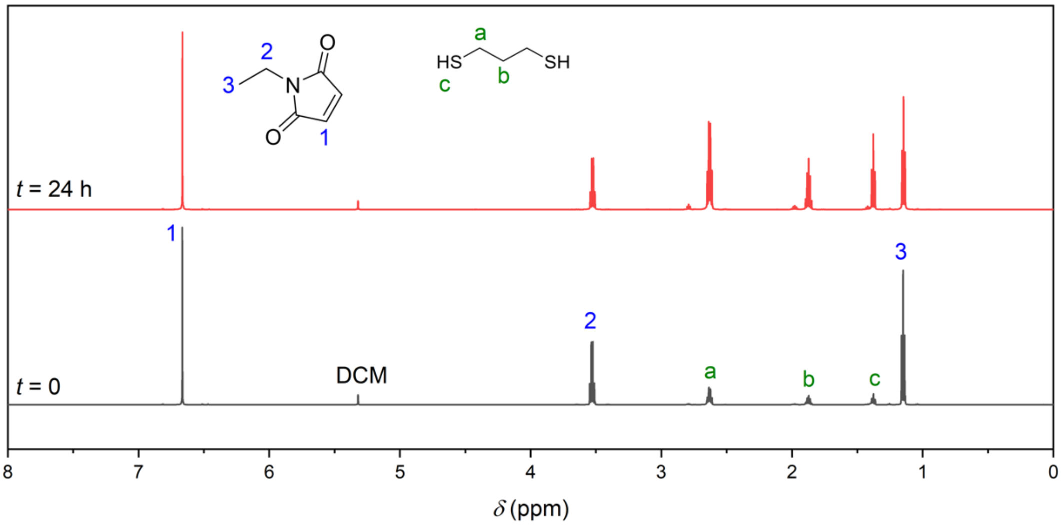

Figure S14. Comparison of ^{1}H NMR spectra of a mixture of *N*-ethylmaleimide and 1,3-propanedithiol in CD_2Cl_2 at t = 0 and after 24 hours at ambient temperature.

8 Orthogonality of Bicyclo[2.2.1]hept-2-ene and *o*-MBA under Irradiation

A solution of 9.30 µmol·mL^{-1} *o*-MBA was mixed with 5.00 eq bicyclo[2.2.1]hept-2-ene in 500 µL deuterated dichloromethane in an NMR tube. The solution was irradiated with the laser tuned to 350 nm as per the general procedure for up to 6 hours. No reaction was observed between *o*-MBA and bicyclo[2.2.1]hept-2-ene (Figure S15).

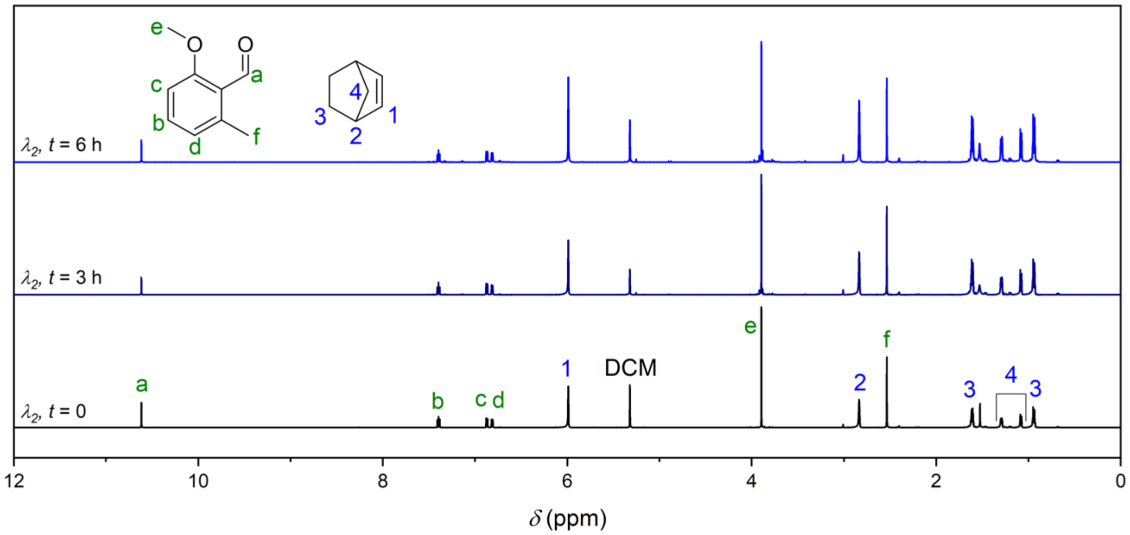

Figure S15. Comparison of ^{1}H NMR spectra of a mixture of *o*-MBA and bicyclo[2.2.1]hept-2-ene in $CDCl_2$ at t = 0, after 3 hours and after 6 hours under λ_2 = 350 nm irradiation.

9 ProtPE Deprotection in Acetonitrile

In this experiment, 0.55 mg ProtPE (9.00 mmol·L^{-1}), 1mol% PC and 3.00 eq NEM were dissolved in 250 µL deuterated dichloromethane or acetonitrile. The reaction mixture was placed in a crimped 0.7 mL photovial by LLG Labware, Lab Logistic Group GmbH (Art. Nr. 4-008202) with a magnetic stir bar and irradiation performed as per the general procedure. The result shows that deprotection in ACN is much slower than DCM, reaching only 7% deprotection while in DCM deprotection reached 40% under the same experimental conditions (Figure S16). In this experiment, 1.00 eq of 1,3,5-trimethoxybenzene was added to the stock solutions in order to provide an NMR standard (resonance at 6.1 ppm in ACN and 6.07 ppm in DCM) to ease the determination of the respective concentrations. The ^{1}H NMR signals used for the determination of the concentration of ProtPE and *o*-MBA during irradiation in ACN are at 7.16 ppm and 7.44 ppm, respectively. The ^{1}H NMR signals used for the determination of the concentration of ProtPE and *o*-MBA during irradiation in DCM were at 2.72 ppm and 2.55 ppm, respectively.

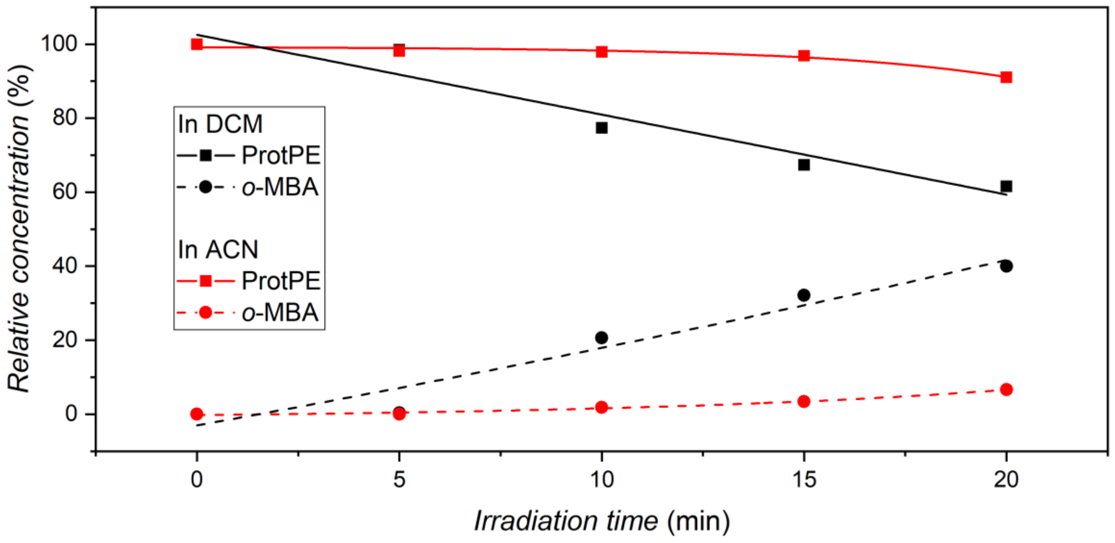

Figure S16. Analysis on the conversion from ProtPE to *o*-MBA under irradiation with λ_1 = 460 nm in different solvents.

10 Cyclisation of *o*-MBA under UV Light Irradiation

10.70 mg of *o*-MBA (3.55 µmol·mL^{-1}) were dissolved in 20 mL DCM and irradiated for 3 hours with a 3 W, 365 nm LED located 1 cm below a round bottom flask (maximum volume = 50 mL) closed with a rubber septum. The reaction was performed under stirring. A low concentration is important for this reaction since, if the concentration is not sufficiently low, dimerization can occur. Analysis of the crude product showed residual *o*-MBA and a large amount of the targeted unknown side-product. The side-product is likely 7-methoxy-1,3-dihydroisobenzofuran-1-ol; which subsequently further degrades to 7-methoxyisobenzofuran-1(3H)-one at elevated temperatures (≈ 40° C). Despite our best effort to minimize the formation of this side-product in the deprotection reaction, this side-product could not be eliminated fully, being present in all experiments where the reaction mixture was irradiated with UV light.

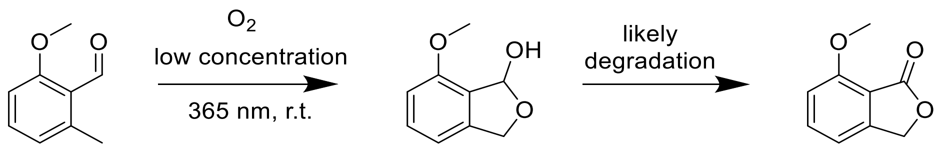

^{1}**H NMR** (600 MHz, Methylene chloride-d_2) δ 7.32 (t, *J* = 7.9 Hz, 1H), 6.86 (d, *J* = 8.3 Hz, 1H), 6.73 (d, *J* = 7.7 Hz, 1H), 6.02 (s, 1H), 5.31 (d, *J* = 15.6 Hz, 1H), 4.88 (d, *J* = 15.5 Hz, 1H), 3.88 (s, 3H).

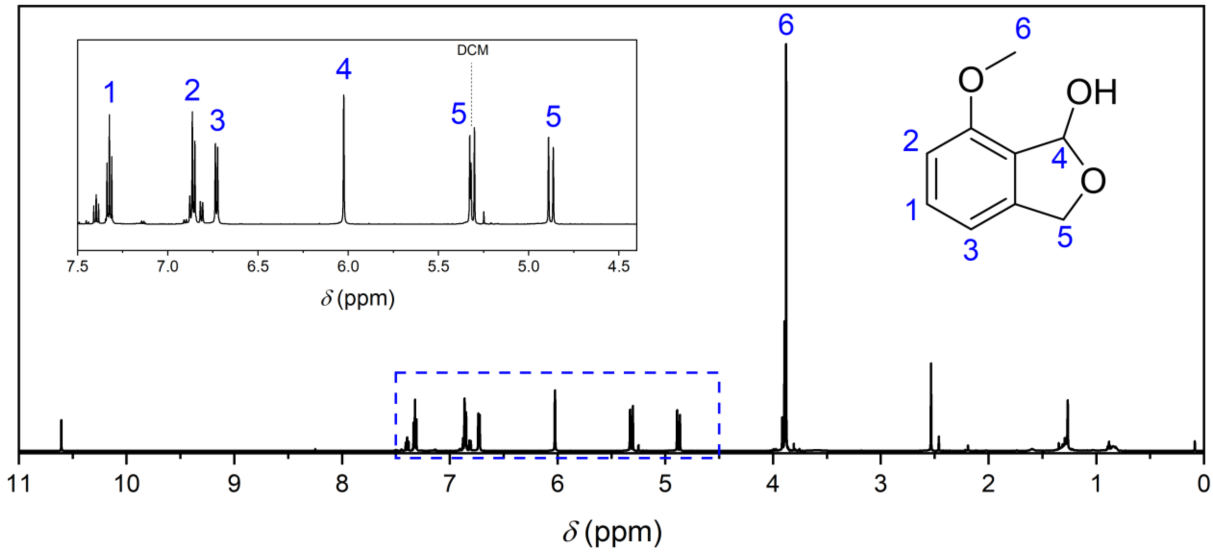

Figure S17. ^{1}H NMR spectrum (600 MHz) of 7-methoxy-1,3-dihydroisobenzofuran-1-ol in CD_2Cl_2. Unassigned peaks belong to the *o*-MBA starting material. This compound has a spectrum similar to comparable derivatives (1,7-dimethoxy-1,3-dihydroisobenzofuran) in the literature.[6]

^{13}C NMR (151 MHz, Methylene chloride-d_2) δ 157.33, 134.25, 130.27, 121.14, 116.67, 109.86, 92.16, 71.23, 56.11.

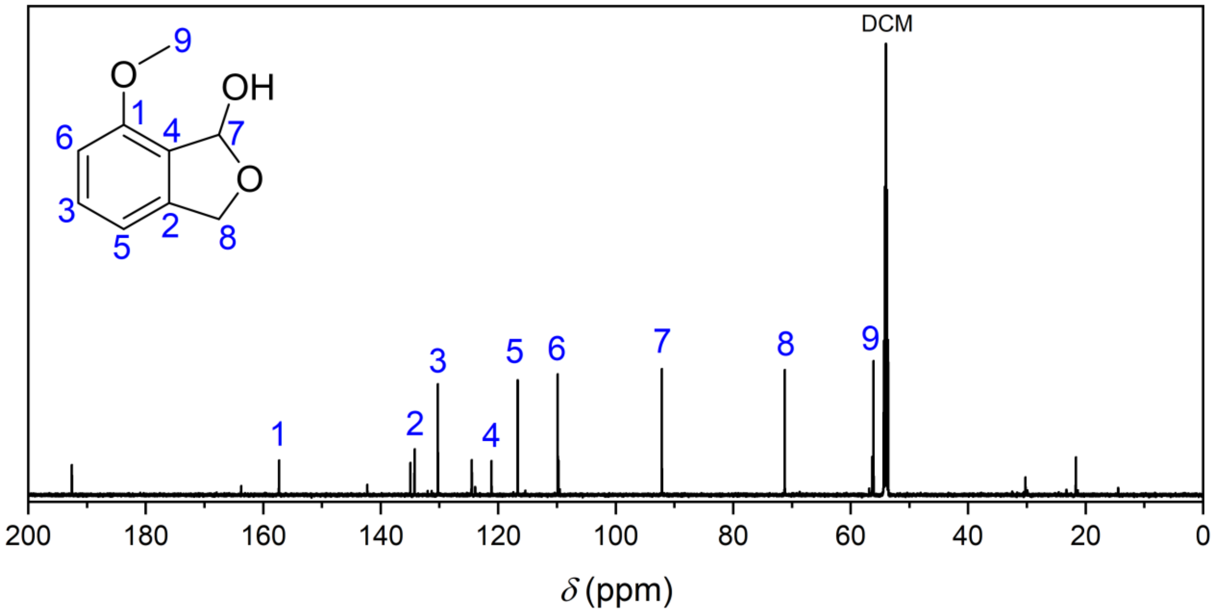

Figure S18. ^{13}C NMR spectrum (151 MHz) of 7-methoxy-1,3-dihydroisobenzofuran-1-ol in CD_2Cl_2. Unassigned peaks belong to the *o*-MBA.

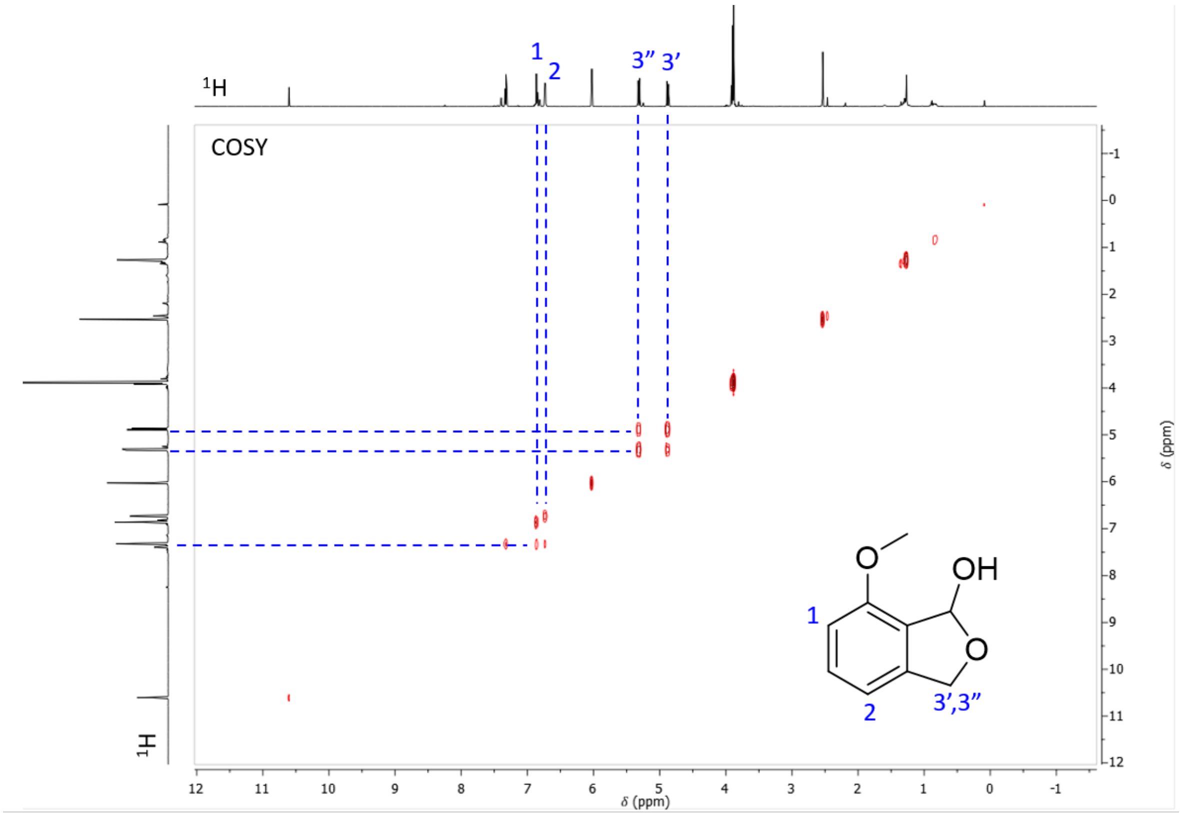

Figure S19. ^{1}H-^{1}H correlated spectroscopy (COSY) spectrum of 7-methoxy-1,3-dihydroisobenzofuran-1-ol in CD_2Cl_2.

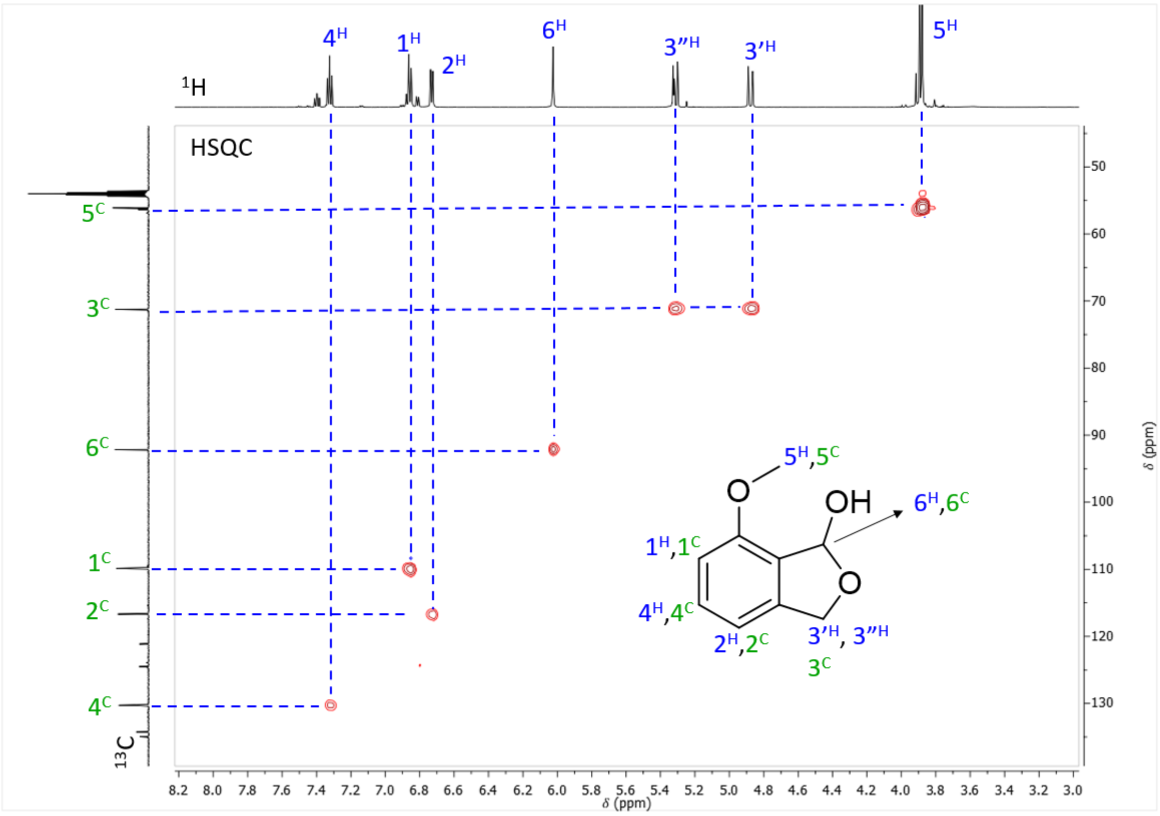

Figure S20. Heteronuclear single quantum coherence spectroscopy (HSQC) spectrum of 7-methoxy-1,3-dihydroisobenzofuran-1-ol in CD_2Cl_2.

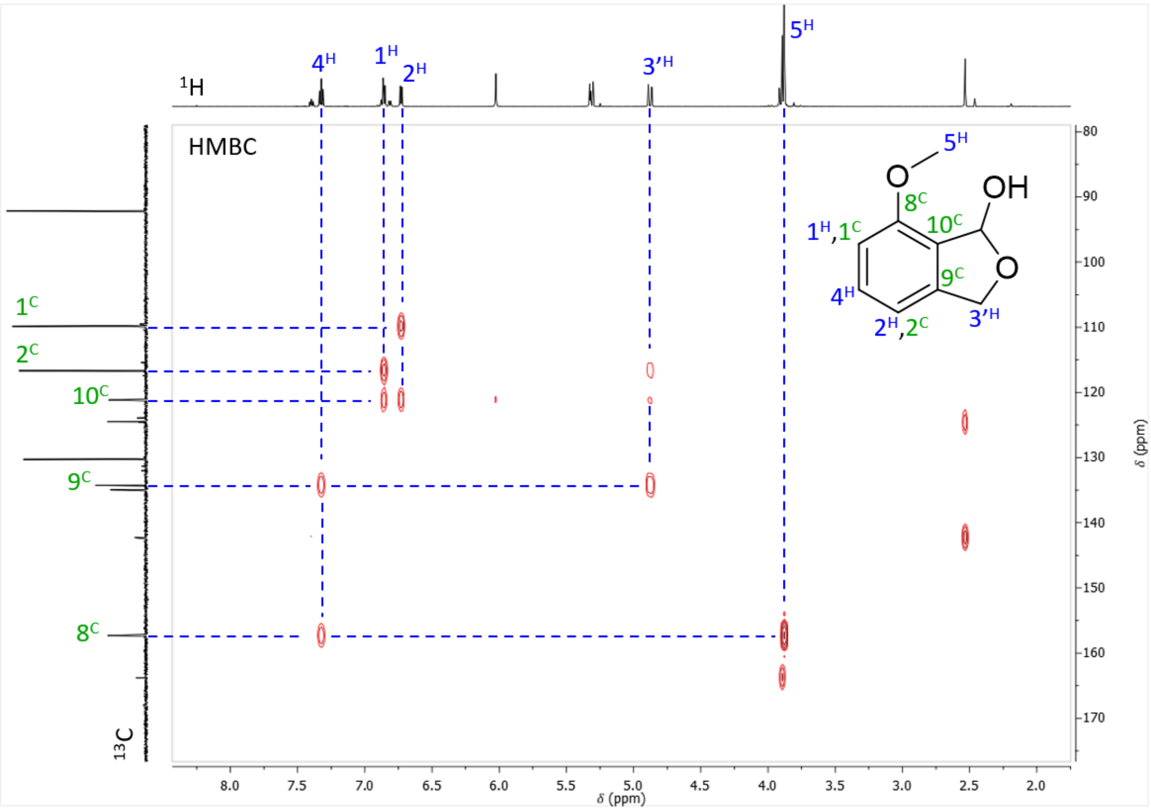

Figure S21. Heteronuclear multiple bond correlation (HMBC) of 7-methoxy-1,3-dihydroisobenzofuran-1-ol in CD_2Cl_2.

The degradation of the side-product to 7-methoxyisobenzofuran-1(3H)-one was inferred by LC-HRMS.

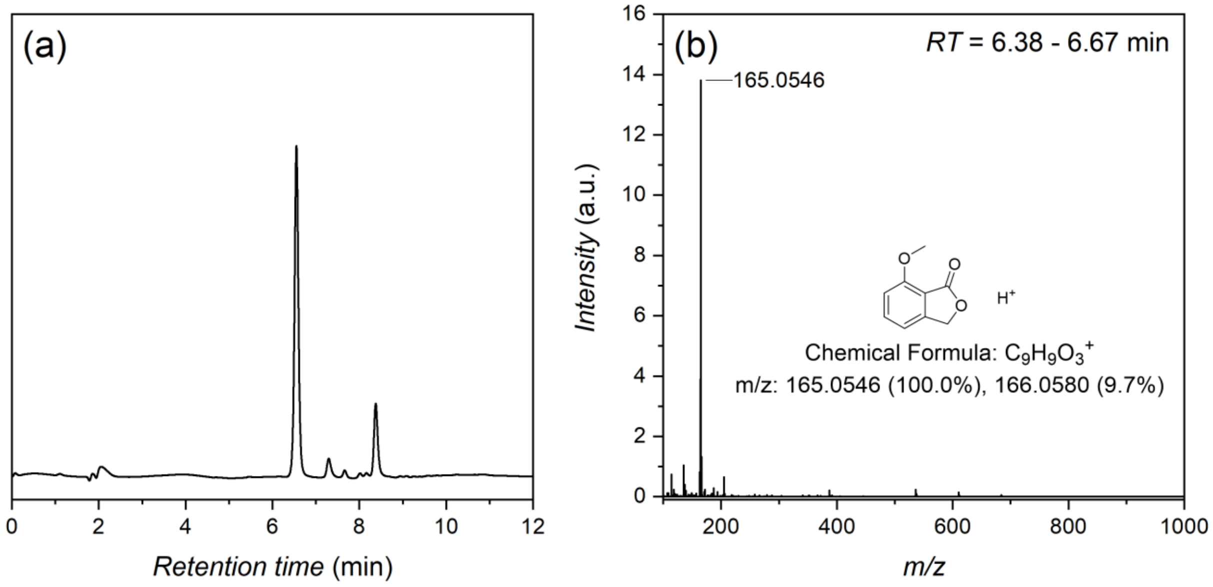

Figure S22. LC-HRMS analysis of 7-methoxyisobenzofuran-1(3H)-one. (a) Chromatogram of the 280 nm UV-Vis channel and (b) accumulated MS spectra at *RT* = 6.38 – 6.67 min with the insert of the chemical structure and its m/z prediction (no deviation).

11 Heat Effect on the Deprotection of ProtPE

Irradiating the standard reaction mixture with a visible light source leads to the formation of a side-product indicated by the star (*) in Figure S23. The aromatic triplet of the side-product overlaps with the aromatic triplet of the D-A adduct (red line in Figure S23). As The distinct peak at 5.8 ppm, indicative for the D-A adduct, is not present in the reaction mixture after irradiation with visible light only, thus, proving that no D-A adduct was formed.

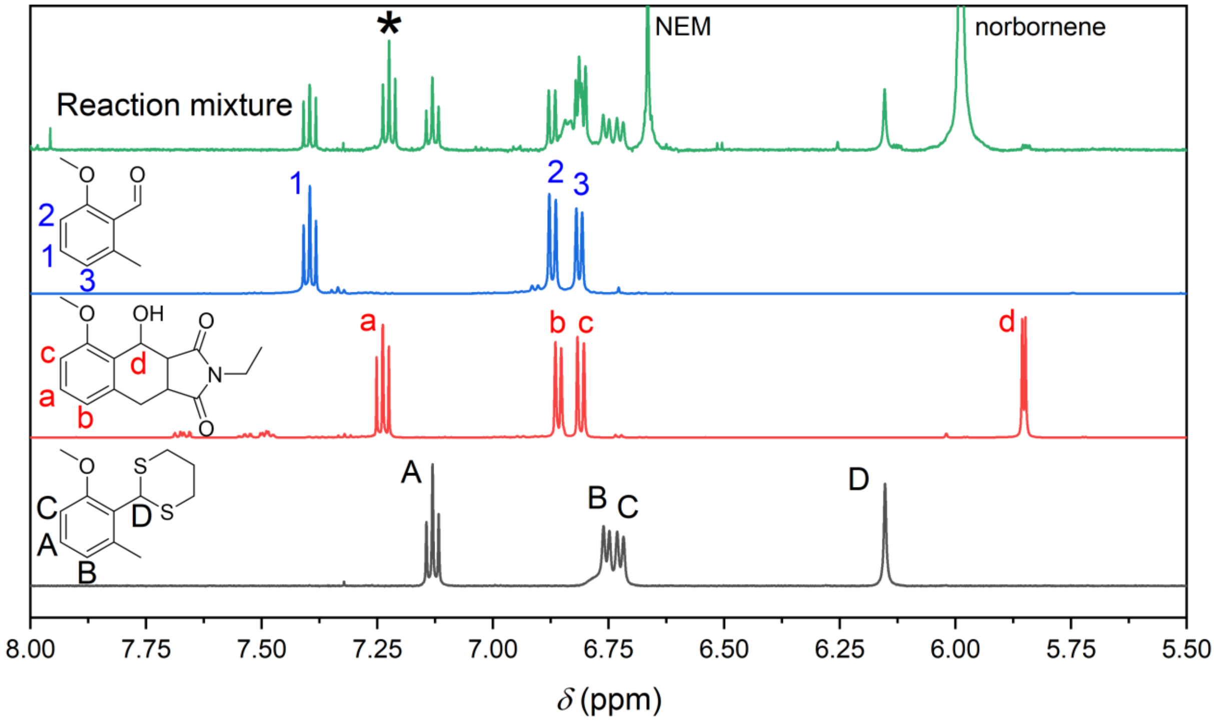

Figure S23. Reaction mixture after irradiation with λ_1 = 430 – 450 nm (10W) following the standard procedure (first from top, green) in comparison with the standard materials *o*-MBA (second from top, blue) and ProtPE (fourth from top, black) and the Diels-Alder adduct (third from top, red).

A significant increase of side-product formation was observed when the visible light source (λ_1) was changed from a 3 W LED to a 10 W LED (Figure S24).

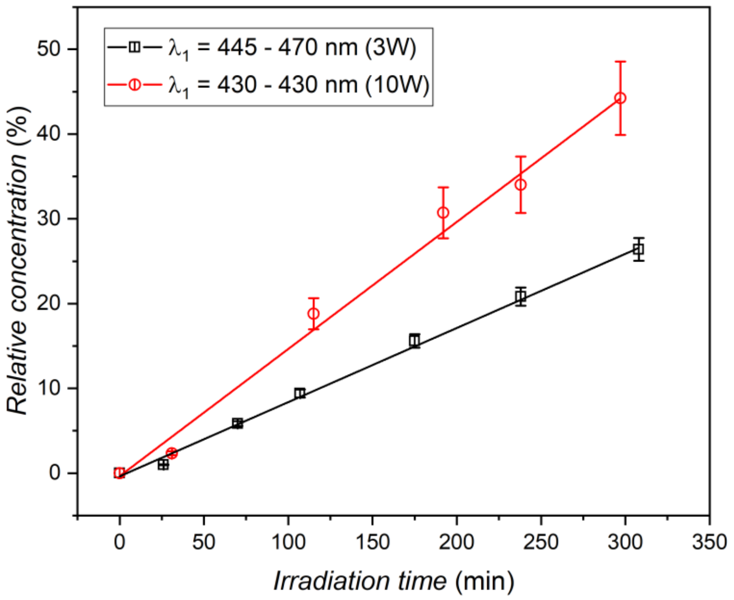

Figure S24. Side-product formation from the deprotection step depending on the light source used.

While isolation of the side-product was not possible, study via LC-HRMS (Figure S25) allowed inferring the oxidation of the sulfur atoms as side-products of the deprotection. Single and double oxidation of the sulfur atoms were observed *via* LC-HRMS. It is believed that upon irradiation, those species can still be deprotected. Therefore, the amount of side products is minimized when the irradiation is performed for a sufficient time and at optimum conditions.

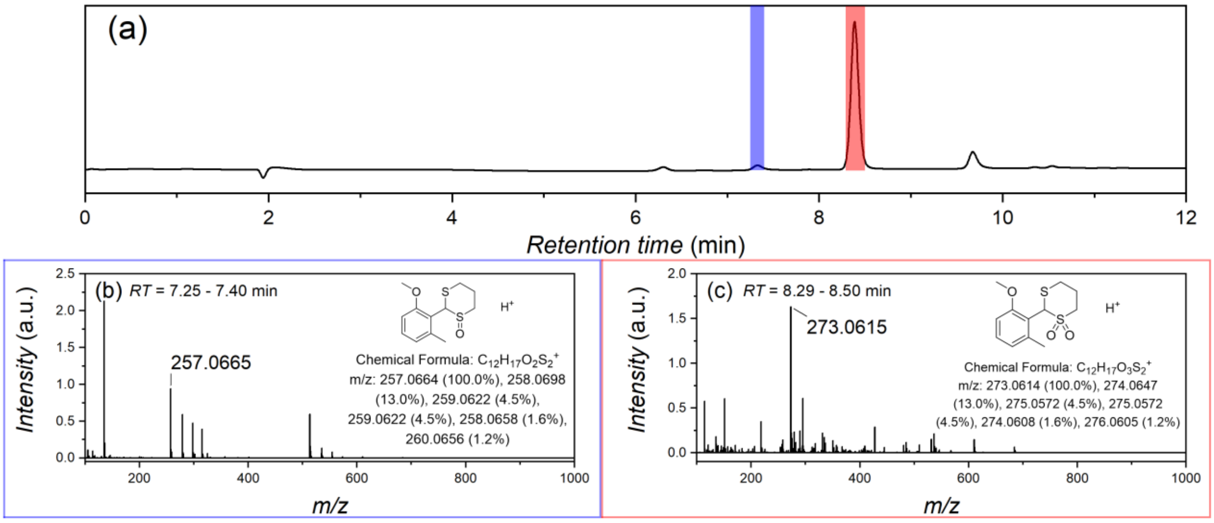

Figure S25. LC-HRMS analysis of the standard reaction mixture after irradiation with λ_1 = 430 – 450 nm (10W) for 300 minutes. (a) Chromatogram of the 280 nm UV-Vis channel with the highlighted areas in blue and red correlating to, respectively, (b) accumulated MS spectra at *RT* = 7.25 – 7.40 min and (c) accumulated MS spectra at *RT* = 8.29 – 8.50 min, both with the insert of the chemical structure and its m/z prediction (both with 0.4 ppm deviation).

12 Full ^{1}H-NMR Spectra of Sequential Irradiation

Diels-Alder adduct formation can be observed under sequential irradiation (Figure S26).

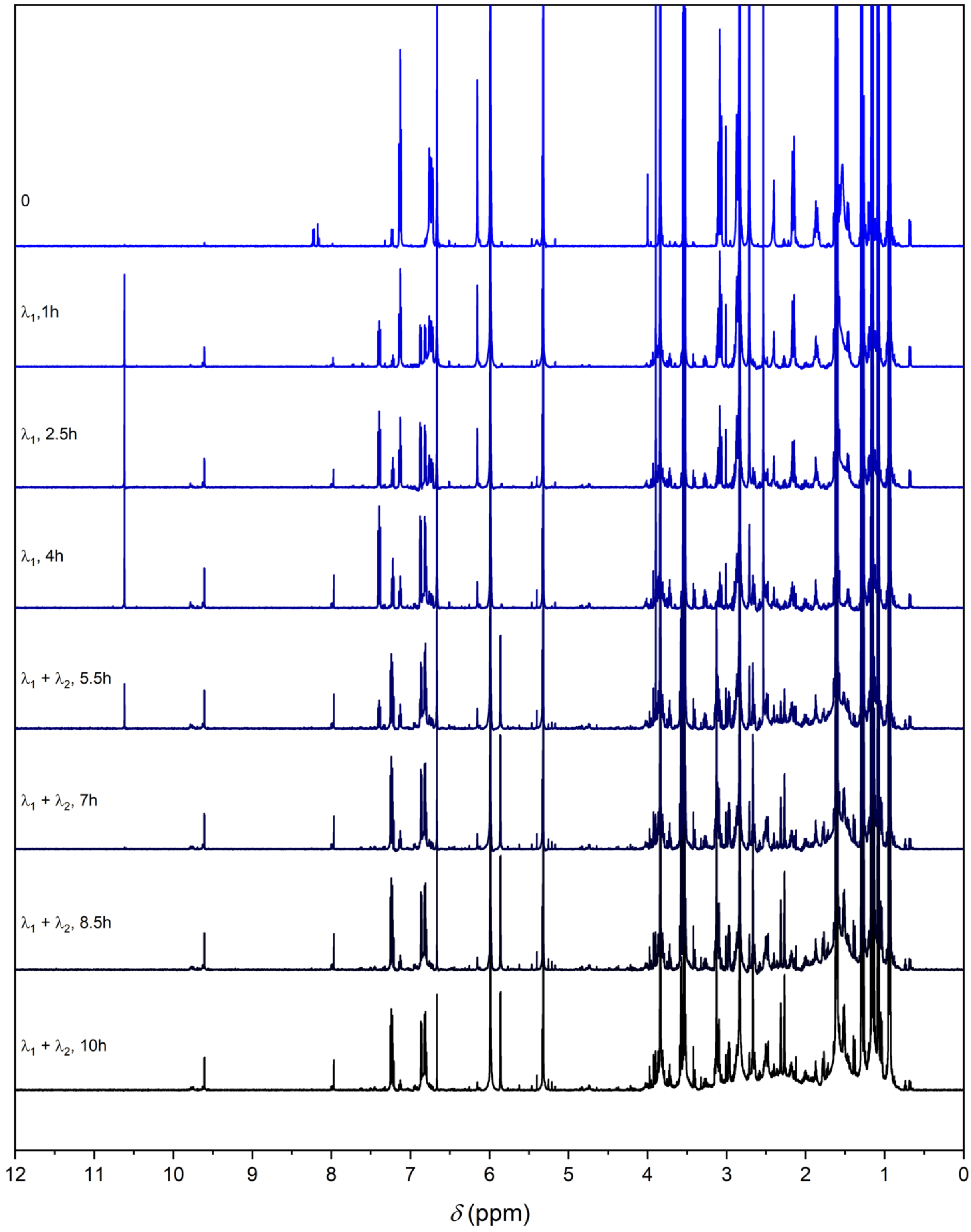

Figure S26. Irradiation study of ProtPE (9.2 mmol·L^{-1}, 1 eq), PC (1mol%), bicyclo[2.2.1]hept-2-ene (5 eq) and NEM (1.3 eq) in 500 µL deuterated DCM with λ_1 = 415 nm subsequently with λ_1 and λ_2 = 350 nm.

13 Maximum Deprotection

In this experiment, 1.76 mg ProtPE (9.20 mmol·L^{-1}, 1.00 eq), 1mol% PC, 1.30 eq NEM and 1.30 eq. bicyclo[2.2.1]hept-2-ene were dissolved in 250 μL deuterated dichloromethane. The reaction mixture was placed in a crimped 0.7 mL photovial by LLG Labware, Lab Logistic Group GmbH (Art. Nr. 4-008202) with a magnetic stir bar and irradiation performed with visible light only as per the general procedure. In this experiment, 0.20 eq of 1,3,5-trimethoxybenzene (TMB) was added to the stock solutions in order to provide an NMR standard (resonance at 6.07 ppm in DCM) to ease the determination of the respective concentrations. The ^{1}H NMR signals used for the determination of the concentration of ProtPE and *o*-MBA during irradiation in DCM were at 2.72 ppm and 2.55 ppm, respectively.

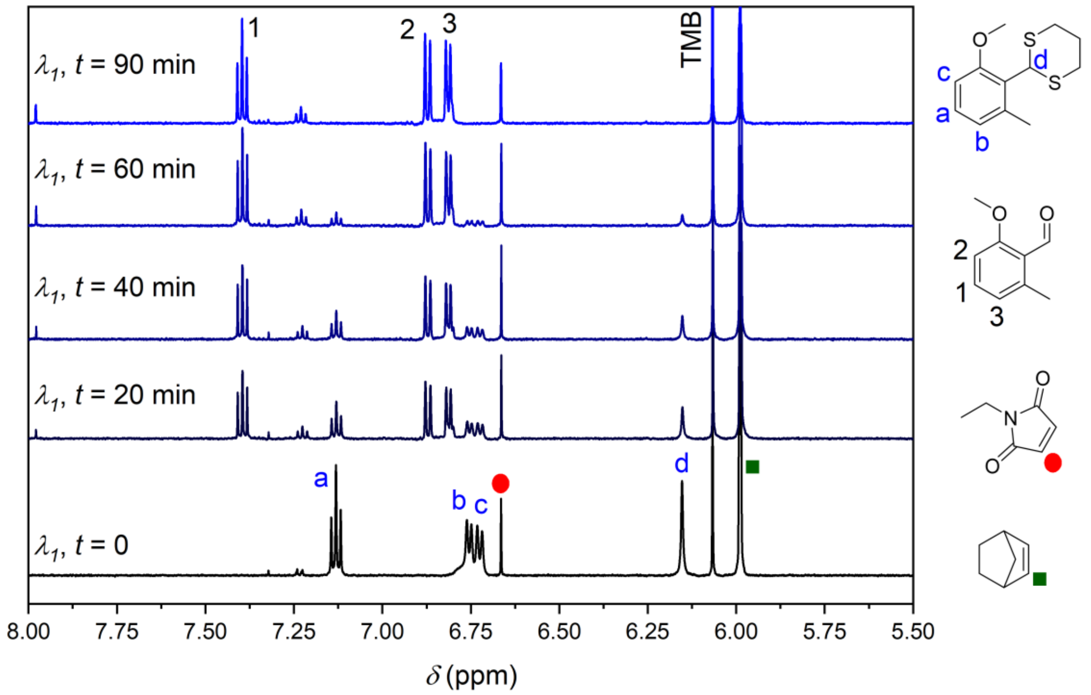

Figure S27. Comparison of ^{1}H NMR spectra of a mixture of ProtPE, PC, NEM, and bicyclo[2.2.1]hept-2-ene in $CDCl_2$ at t = 0, after 20 min, 40 min, 60 min and 90 min under λ_1 = 460 nm irradiation.

ProtPE was fully consumed within 1 hour, affording 86 % *o*-MBA and 14 % partial oxidation of the sulfur atoms.

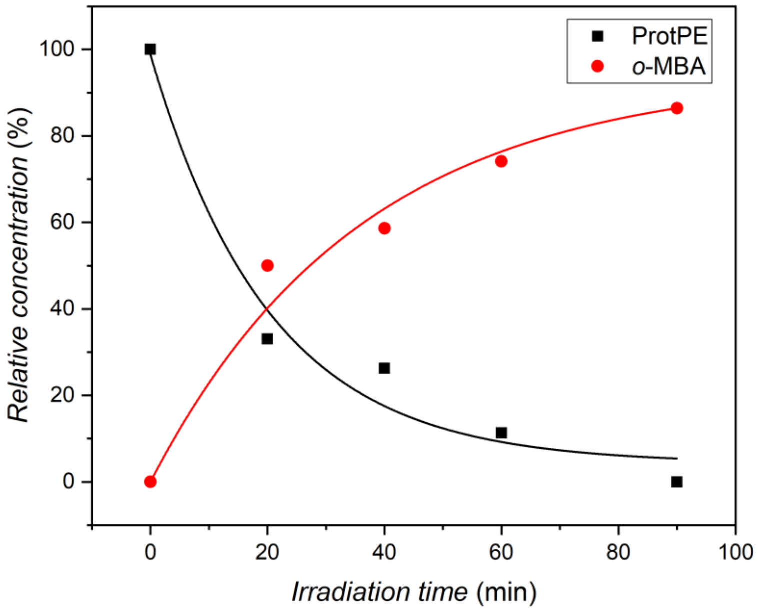

Figure S28. Analysis on the conversion from ProtPE to *o*-MBA under irradiation with λ_1 = 460 nm in deuterated DCM.

14 Higher Diels-Alder Adduct Formation Yield

In this experiment, 0.35 mg *o*-MBA (9.38 mmol·L^{-1}, 1.00 eq), 1mol% PC and 3.03 eq NEM were dissolved in 250 μL deuterated dichloromethane. The reaction mixture was placed in a crimped 0.7 mL photovial by LLG Labware, Lab Logistic Group GmbH (Art. Nr. 4-008202) with a magnetic stir bar and irradiation performed with UV light only as per the general procedure. In this experiment, 0.93 eq of 1,3,5-trimethoxybenzene (TMB) was added to the stock solutions in order to provide an NMR standard (resonance at 6.07 ppm in DCM) to ease the determination of the respective concentrations. The ^{1}H NMR signals used for the determination of the concentration of *o*-MBA and Diels-Alder adduct during irradiation in DCM were at 7.41 ppm and 7.26 ppm, respectively. The conversion to the Diels-Alder product was 98 %. The conversion was determined by the beforementioned ^{1}H NMR signals and the internal standard.

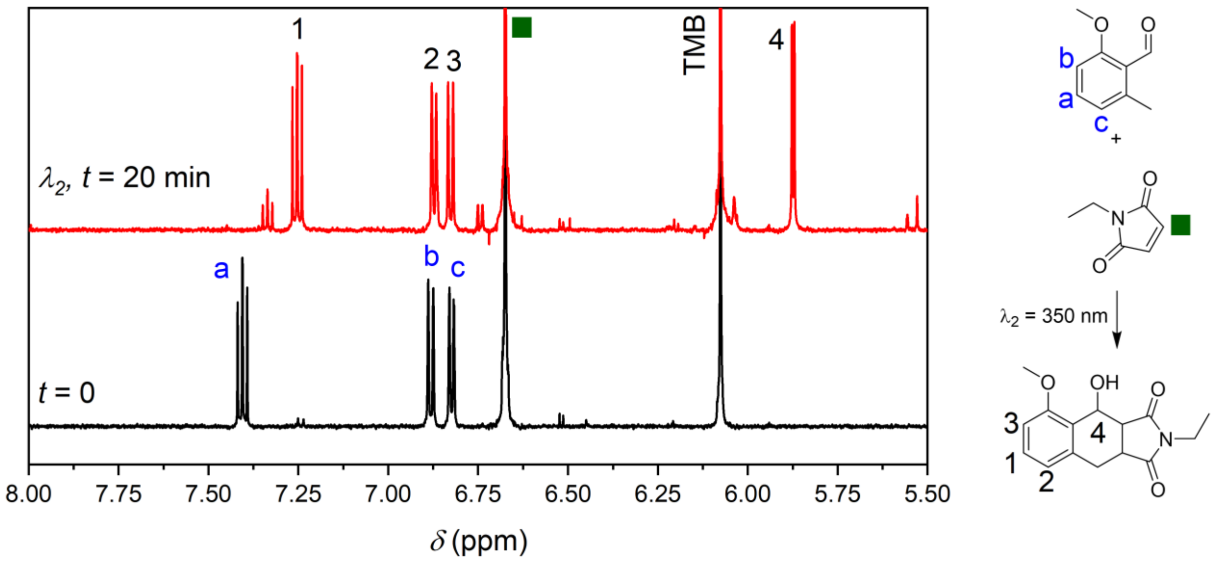

Figure S29. Comparison of ^{1}H NMR spectra of a mixture of *o*-MBA, PC and NEM in CD_2Cl_2 at t = 0 and after 20 min under λ_2 = 350 nm irradiation.

15 References

(1) Martiny, M.; Steckhan, E.; Esch, T. Cycloaddition Reactions Initiated by Photochemically Excited Pyrylium Salts. *Chem. Ber.* **1993**, *126* (7), 1671–1682. https://doi.org/10.1002/cber.19931260726.

(2) Konrad, W.; Bloesser, F. R.; Wetzel, K. S.; Boukis, A. C.; Meier, M. A. R.; Barner-Kowollik, C. A Combined Photochemical and Multicomponent Reaction Approach to Precision Oligomers. *Chem. - A Eur. J.* **2018**, *24* (14), 3413–3419. https://doi.org/10.1002/chem.201705939.

(3) Menzel, J. P.; Noble, B. B.; Lauer, A.; Coote, M. L.; Blinco, J. P.; Barner-Kowollik, C. Wavelength Dependence of Light-Induced Cycloadditions. *J. Am. Chem. Soc.* **2017**, *139* (44), 15812–15820. https://doi.org/10.1021/jacs.7b08047.

(4) Mandal, S. K.; Roy, S. C. Titanocene(III) Chloride Mediated Radical-Induced Synthesis of 3,4-Dihydroisocoumarins: Synthesis of (±)-Hydrangenol, (±)-Phyllodulcin, (±)-Macrophyllol and (±)-Thunberginol G. *Tetrahedron* **2008**, *64* (49), 11050–11057. https://doi.org/10.1016/j.tet.2008.09.075.

(5) Firouzabadi, H.; Iranpoor, N. Iodine Catalyzes Efficient and Chemoselective Thioacetalization of Carbonyl Functions , Transthioacetalization of O , O - and S , O -Acetals and Acylals Protections of Carbonyl Groups as Thioacetals Are Quite Often a Necessary Requirement in the Synthesis. **2001**, No. c, 7527–7529. https://doi.org/10.1021/jo015798z.

(6) Egan, B. A.; Paradowski, M.; Thomas, L. H.; Marquez, R. Regiocontrolled Rearrangement of Isobenzofurans. *Org. Lett.* **2011**, *13* (8), 2086–2089. https://doi.org/10.1021/ol200498k.
